# Proteomics analysis to reveal biological pathways and predictive proteins in the survival of high-grade serous ovarian cancer

**DOI:** 10.1038/s41598-017-10559-9

**Published:** 2017-08-29

**Authors:** Hongyu Xie, Wenjie Wang, Fengyu Sun, Kui Deng, Xin Lu, Huijuan Liu, Weiwei Zhao, Yuanyuan Zhang, Xiaohua Zhou, Kang Li, Yan Hou

**Affiliations:** 1 Department of Epidemiology and Biostatistics, School of Public Health, Harbin Medical University, Harbin, 150086 China; 2 Department of Cardiology, the First Affiliated Hospital of Harbin Medical University, Cardiovascular Institute, Harbin Medical University, Harbin, China; 3 School of Life Science and Technology, Harbin Institute of Technology, Harbin, China; 4 Department of Biostatistics, University of Washington, Seattle, 96596 USA

## Abstract

High-grade serous ovarian cancer (HGSC) is an aggressive cancer with a worse clinical outcome. Therefore, studies about the prognosis of HGSC may provide therapeutic avenues to improve patient outcomes. Since genome alteration are manifested at the protein level, we integrated protein and mRNA data of ovarian cancer from The Cancer Genome Atlas (TCGA) and Clinical Proteomic Tumor Analysis Consortium (CPTAC) and utilized the sparse overlapping group lasso (SOGL) method, a new mechanism-driven variable selection method, to select dysregulated pathways and crucial proteins related to the survival of HGSC. We found that biosynthesis of amino acids was the main biological pathway with the best predictive performance (AUC = 0.900). A panel of three proteins, namely EIF2B1, PRPS1L1 and MAPK13 were selected as potential predictive proteins and the risk score consisting of these three proteins has predictive performance for overall survival (OS) and progression free survival (PFS), with AUC of 0.976 and 0.932, respectively. Our study provides additional information for further mechanism and therapeutic avenues to improve patient outcomes in clinical practice.

## Introduction

Epithelial ovarian cancer (EOC) is composed of four major histologic subtype: serous, clear cell, endometrioid, and mucinous adenocarcinomas. Among them, high-grade serous ovarian cancer (HGSC), accounting for approximately 70% of EOC^1^, is an aggressive ovarian cancer that associated with a worse clinical outcome^2^. Despite initial aggressive treatment, patients always have an extremely poor overall survival (OS) with the 5-year survival rate less than 40%^3, 4^. The underlying biological characteristics relevant to the prognosis of ovarian cancer still remain unclear and thereby present the challenge of explaining how molecular alterations drive cancers.

With the development of microarray technologies, studies about genetic markers and gene expression profiles have sought to elucidate the molecular determinant of outcome in serous ovarian cancers^5–7^. However, alterations observed at the genome levels are manifested at the protein level, because proteins link genotypes to phenotypes. Although most previous studies have been used to explore the association between specific proteins and prognosis of ovarian cancer^8–10^, cancer is a heterogeneity disease that does not only involve individual molecule but also combination of molecules associated with the processes of cancer. Yang *et al*. identified nine protein markers significantly associated with progression free survival (PFS) based on the least absolute shrinkage and selection operator (lasso) and constructed a protein-driven index of ovarian cancer (PROVAR) scores to predict the recurrence time for ovarian cancer patients^11^. However, Zhang *et al*. performed an external validation in 67 patients and found that the PROVAR signature was prognosis of survival (Benjamini-Hochberg adjusted *p* value = 0.11). Meanwhile, Zhang *et al*. utilized *trans*-affected protein data from the most influential copy number alterations (CNAs) (four altered regions on chromosomes 2, 7, 20 and 22) to build a model to predict the overall survival^12^. However, the predictive performance and clinical practicability of the model were not validated by other studies, and it deserved further study to explore OS of ovarian cancer from the perspective of protein and mRNA convergence systematically. The most important goal of cancer survival is to identify the dysregulated molecular pathways and individual molecule to reveal the mechanism of cancer and develop the effective treatment. Although univariate cox regression and lasso are effective in identifying signatures associated with the prognosis of cancer patients^13–15^, these methods seldom combined biological information to select biomarkers, thereby it is one of the reasons that these biomarkers are not widely used in clinical practice. Although in recent years, network-based biomarker selection methods have been proposed^16, 17^, these methods would lead to overfitting when the predictive model included all selected molecules from network analysis^18^. Therefore, biomarker selection based on a priori biological pathway knowledge, especially in the condition that overlapped variables across pathways and in line with the realities needed.

In this paper, we integrated protein and mRNA data of ovarian cancer from The Cancer Genome Atlas (TCGA) and Clinical Proteomic Tumor Analysis Consortium (CPTAC) and characterized HGSC based on the common information from mRNA to protein. In addition, the sparse overlapping group lasso (SOGL) method^18^, a mechanism-driven biomarker selection method, was utilized to select the main biological pathways and crucial proteins related to OS and further identified predictive proteins for OS in ovarian cancer patients. Meanwhile, we constructed a protein-driven biomarker risk score to predict OS and PFS in HGSC. Prognosis analysis of biological pathways could provide basis for further mechanism research, and selected biomarkers of OS could provide molecule-targeted treatment and improve patient outcomes.

## Results

### Proteome-genome analysis of TCGA HGSC samples

HGSC and clinical data from 169 patients were analyzed at two independent centers, JHU (n = 119) and PNNL (n = 82). 32 samples were analyzed at both centers and utilized to correct the batch effects between two sites, and merged them into a single dataset prior to analysis^12^. In order to present the comprehensive understanding of the information from mRNA to protein, we integrated the proteomics and genomics characterization of HGSC and 3,329 unique proteins paired proteome-genome were used to further analysis. The median OS time of patients was 34.4 months (range, 0.3–182.7 months) and median PFS time of patients was 15.6months (range, 0.3–182.7 months) in this study.

### KEGG enrichment analysis for proteins

We firstly matched the corresponding KEGG-IDs for 3,329 proteins and mapped the KEGG-IDs of the proteins to the pathways using KEGG PATHWAY. In total, 3,259 proteins were enriched to 284 KEGG pathways. A total of 75 pathways were potentially involved in the OS of ovarian cancer with an adjusted pathway false discovery rate (fdr) < 0.05 (see Supplementary Table S1). The number of proteins in each significant pathway ranged from 11 to 450.

### Identification of the dysregulation pathways and crucial proteins

**Figure 1:**
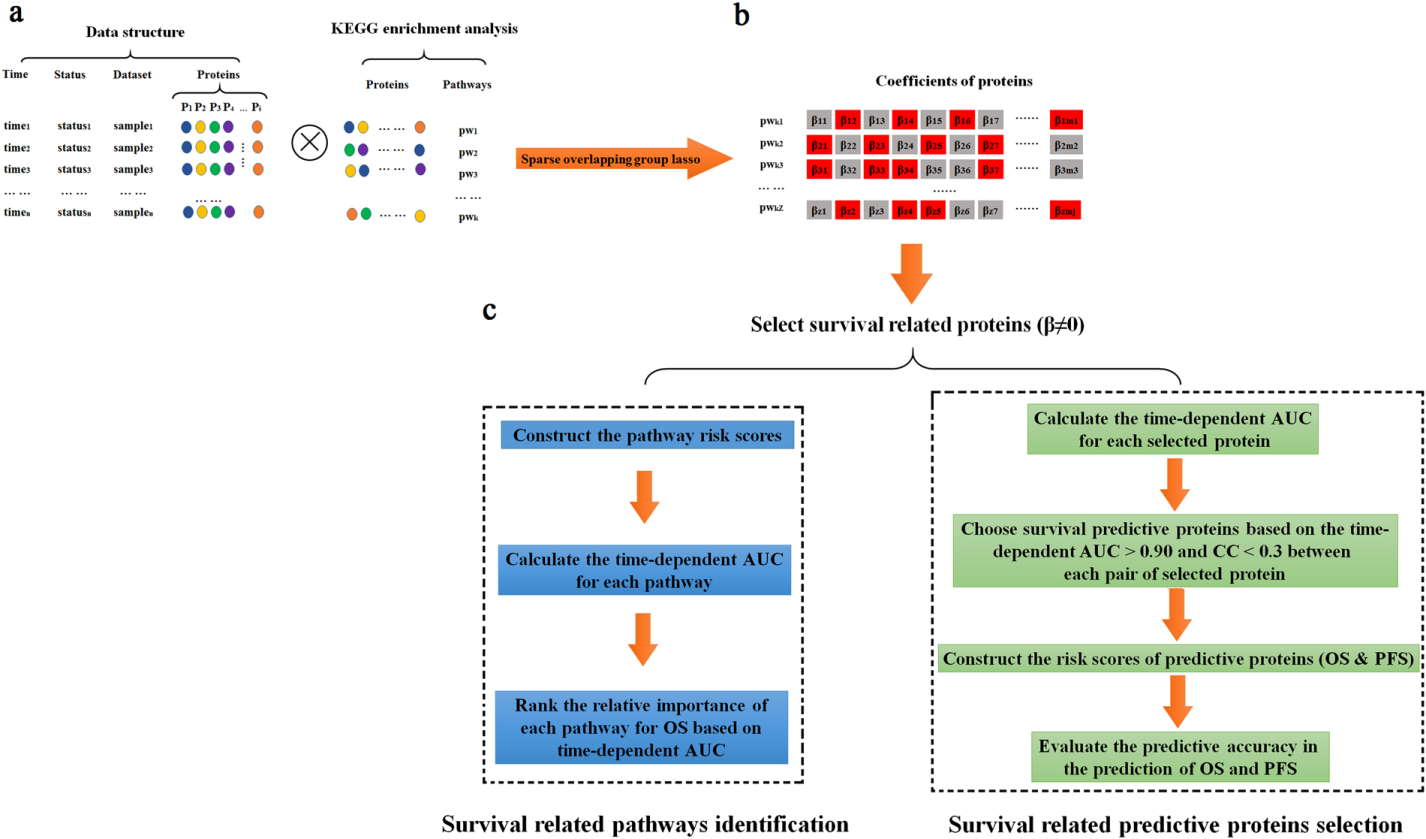
The overview workflow of the analysis process. (**a**) Subdivision of proteins into groups based on KEGG database (**b**) Selection of dysregulated biological pathways and crucial proteins related to the OS of ovarian cancer based on SOGL method. The coefficients of proteins with gray shadow were estimated to zero; The coefficients of proteins with red shadow were estimated to nonzero, these proteins defined as crucial proteins related to the OS and pathways of them were defined as dysregulated biological pathways. (**c**) Survival related pathway identification and survival related predictive proteins selection.

We utilized the SOGL to identify the key pathways and crucial proteins associated with the OS based on the results of KEGG enrichment analysis. Schematic diagram of SOGL was presented in Supplementary Fig. S1. Since nonzero coefficient for each feature and its involved pathway based on SOGL were thought to be associated with OS of HGSC. Although 455 proteins across 75 pathways associated with OS of HGSC (see Supplementary Table S2, Fig. S2) have been identified, 200 proteins are across at least two pathways. The remaining 250 proteins appear in 50 pathways as the potential biomarkers. We hypothesized that proteins across pathways might be indicators of key regulators with strong impact on OS. As an example, the protein MAPK13 participants in seven different pathways, which might be an important indicator. The analysis process was presented in Fig. 1.

### Pathways associated with overall survival

**Figure 2:**
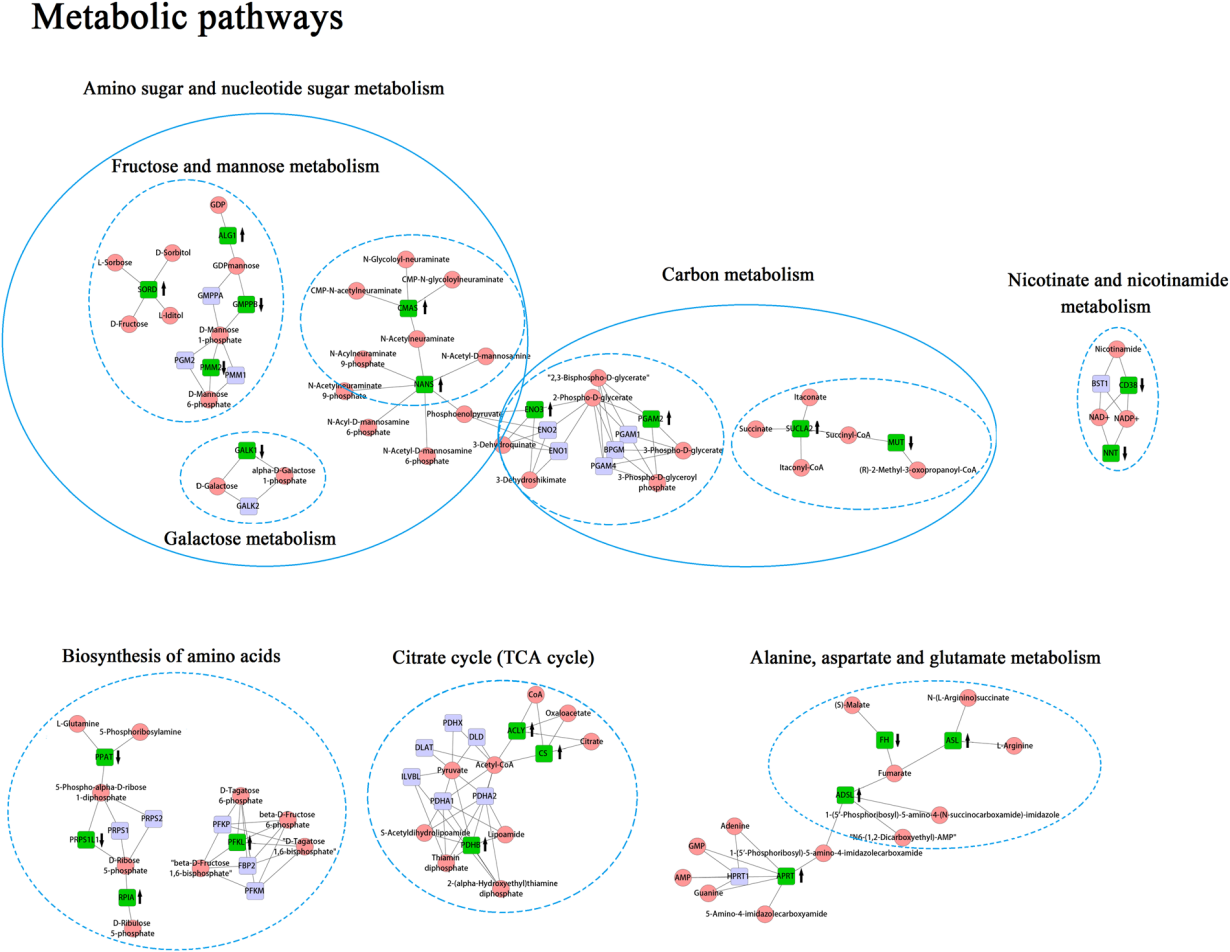
Overall survival related protein sub-pathways involved in Metabolic pathways. Green boxes were selected proteins and red nodes were the protein corresponding compounds, such as metabolites. ↑ Represented the coefficients of proteins <0 and indicated highly expressed with the prolongation of the survival time;↓ Represented the coefficients of proteins > 0 and indicated lowly expressed with the prolongation of the survival time.

**Figure 3:**
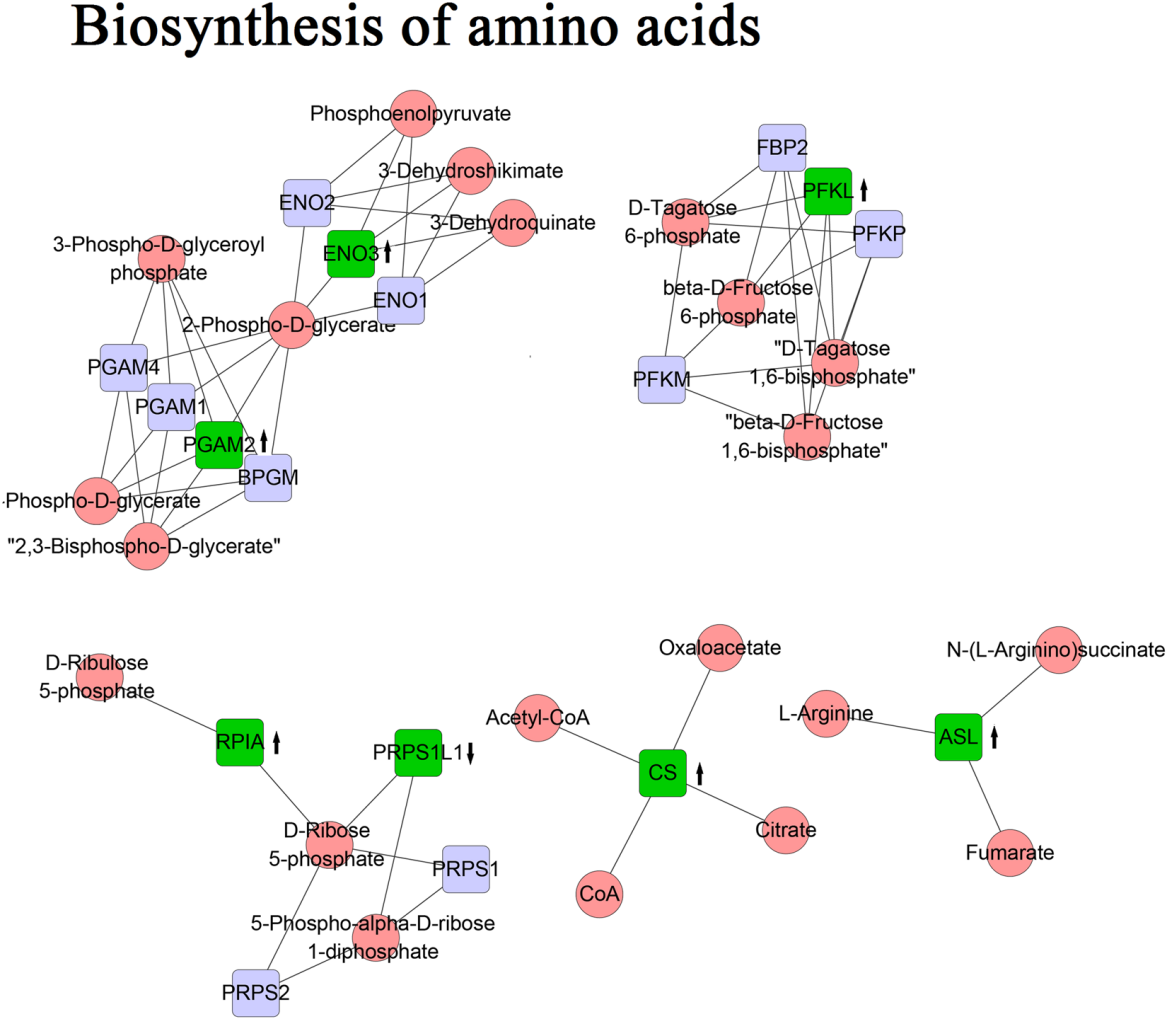
Overall survival related proteins involved in biosynthesis of amino acids.

To gain better insight into the protein interactions that affect clinical outcome, we constructed a relative pathway score for each pathway, which defined as a linear combination of the proteins in each pathway and the coefficients subjected to the SOGL coefficients. Time-dependent AUC^19^ was utilized to evaluate the predictive accuracy of 10-year survival of ovarian cancer patients for each pathway and the results were listed in Supplementary Table S3. We found metabolic pathways played important roles to the prognosis of ovarian cancer (Fig. 2). Biosynthesis of amino acids pathway, one of the metabolic pathways, was defined as a main biological pathway related to the OS of ovarian cancer with time-dependent AUC of 0.90 (Fig. 3).

### Predictive proteins and a protein-driven risk score

Three proteins, namely as EIF2B1, PRPS1L1 and MAPK13 were selected as potential predictive proteins based on univariate AUC > 0.90 (see Supplementary Table S3) and spearman correlation coefficient (CC) < 0.30 between each pair of proteins. The protein-driven risk score, a linear combination of three proteins, was displayed as follows and the coefficient for each protein was the weight in Cox-regression:

$$\begin{array}{c}{\rm{Predictive}}\,{\rm{risk}}\,{{\rm{score}}}_{{\rm{OS}}}=(-0.947\times {\rm{EIF}}2B1)+(-0.623\times {\rm{PRPS}}1L1)\\ \quad \quad \quad \quad \quad \quad \quad \quad \quad \,\,\,+(-0.578\times {\rm{MAPK}}13)\end{array}$$

$$\begin{array}{c}{\rm{Predictive}}\,{\rm{risk}}\,{{\rm{score}}}_{{\rm{PFS}}}=(-0.384\times {\rm{EIF}}2B1)+(-0.260\times {\rm{PRPS}}1L1)\\ \quad \quad \quad \quad \quad \quad \quad \quad \quad \quad \,\,\,+(-0.675\times {\rm{MAPK}}13)\end{array}$$

where protein expression is scaled.

**Figure 4:**
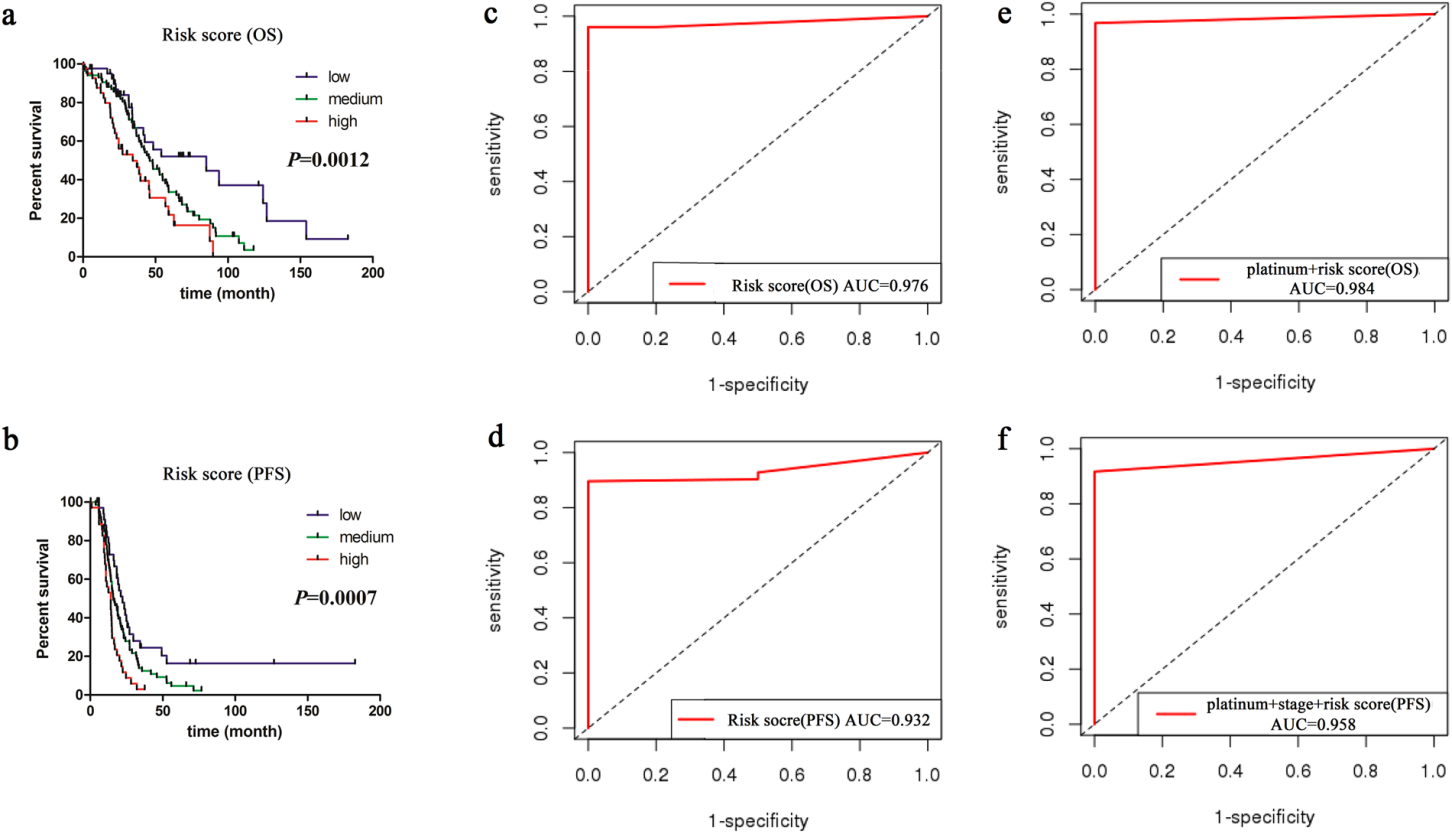
Evaluation of predictive performance of risk score. (**a**,**b**) Kaplan-Meier curve and log-rank test among low, medium and high expression groups for protein-driven risk scores (a: OS, b: PFS). (**c**,**d**) Time-dependent ROC curves evaluating predictive accuracy of ten-year overall survival based on protein-driven risk scores (c: OS, d: PFS). (**e**,**f**) Time-dependent ROC curves evaluating predictive accuracy of ten-year overall survival based on the comprehensive predictive risk score combined the protein-driven predictive risk score with clinical factors (e: OS, f: PFS).

Kaplan-Meier analysis and log-rank test were performed to compare the discriminant capability of predictive risk score for OS and PFS among low, medium, and high risk groups (*P* = 0.0012 and *P* = 0.0007) (Fig. 4a,b).

### Predictive performance of the risk score for HGSC

To test whether the risk score was an independent predictor for HGSC, both univariate and multivariate analysis were performed using a Cox proportional hazards model with the predictive risk score and clinical factors. Factors with univariate analysis *P* value < 0.05 were used for further multivariate analysis. The multivariate analysis results for OS and PFS were presented in Table 1 (OS: risk score (*P* = 2 × 10^−4^), stage (*P* = 0.300) and Platinum status (*P* = 6.5 × 10^−11^)) and Table 2 (PFS: risk score (*P* = 0.010), stage (*P* = 0.022) and Platinum status (*P* < 2 × 10^−16^)). Predictive risk score and platinum status were consistently significant for both OS and PFS regardless of univariate or multivariate analysis. Pathological stage was the only significant for PFS, but not with OS. Ovarian cancer patients sensitive to the platinum chemotherapy would live longer or had a longer PFS time compared with those who were resistant, and the higher the stage was, the shorter the PFS time was.

**Table 1:** Univariate and multivariate Cox regression analysis of risk score alone and clinical factors associated with overall survival.

Factors	*P* value	*HR*	95% *CI*
Univariate analysis			
Risk score	6.5 × 10^−5^	***2.714***	1.661–4.432
Age (vs. ≤50 > 50 y)	0.093	1.559	0.929~2.618
Stage (I vs. II vs. III vs. IV)	0.031	1.523	1.041~2.230
Tumor residual (No macroscopi disease vs. 1~10mm vs. 11~20 mm vs. > 20 mm)	0.990	0.999	0.823~1.214
Platinum status (Resistant vs. Sensitive)	6.1 × 10^−11^	0.193	0.118~0.316
Multivariate analysis			
Risk score	2.0 × 10^−4^	***2.983***	1.677–5.307
Stage (I vs. II vs. III vs. IV)	0.300	1.242	0.822–1.876
Platinum status (Resistant vs. Sensitive)	6.5 × 10^−11^	0.179	0.106–0.299

Abbreviations: versus (vs); Hazard ratio (*HR*); Confidence interval (*CI*).

**Table 2:** Univariate and multivariate Cox regression analysis of risk score alone and clinical factors associated with progression free survival.

Factors	*P* value	*HR*	95% *CI*
Univariate analysis			
Risk score	1.3 × 10^-3^	***2.702***	1.474–4.953
Age (vs. ≤50 > 50 y)	0.220	1.310	0.815~2.019
Stage (I vs. II vs. III vs. IV)	0.008	1.602	1.131~2.268
Tumor residual (No macroscopi disease vs. 1~10 mm vs. 11~20 mm vs. > 20 mm)	0.250	1.103	0.933~1.304
Platinum status (Resistant vs. Sensitive)	<2 × 10^-16^	0.066	0.038~0.117
Multivariate analysis			
Risk score	0.010	***2.456***	1.237–4.878
Stage (I vs. II vs. III vs. IV)	0.022	1.533	1.063–2.211
Platinum status (Resistant vs. Sensitive)	<2 × 10^-16^	0.072	0.041–0.128

Abbreviations: versus (vs); Hazard ratio (*HR*); Confidence interval (*CI*).

We explored the predictive performance of protein-driven risk score alone and together with clinical factors, separately. The results indicated that the protein-driven risk score had a great predictive accuracy for 10-year OS of ovarian cancer with an AUC value of 0.976 (Fig. 4c). The protein-driven predictive risk score with platinum status achieved a time-dependent AUC value of 0.984 (Fig. 4e), which significantly improved the predictive performance of platinum alone (AUC = 0.645). In addition, we further evaluated the predictive capability of protein-driven risk score alone and together with clinical factors (platinum status and pathological stage) to the PFS for ovarian cancer with AUC values of 0.932 (Fig. 4d) and 0.958 (Fig. 4f), separately. These results indicated that the utility of combination of our predictive proteins and clinical factors improved prognosis predictive accuracy.

## Discussion

It is known that prediction of therapy outcome may provide therapeutic avenues to improve patient outcomes. Due to the heterogeneity of clinical outcomes in ovarian cancer patients, it is urgent to explore the outcome-related molecular signatures, that could provide additional information for molecular mechanism and prognosis prediction. In this study, we integrate the proteomic and genomic profiles of HGSC to systematically identify potential pathways and proteins for predicting therapy outcome. Biosynthesis of amino acids and three potential predictive proteins are found to have excellent predictive performance in the prognosis of ovarian cancer. Our study extended our understanding about molecular mechanism of ovarian cancer from protein level and might provide molecule-targeted treatment to improve patient outcomes in clinical practice.

Previous studies have showed that there was association between the prognosis and specific proteins. Lokman *et.al*. showed high stromal annexin A2 immunostaining was significantly associated with reduced PFS (*P* = 0.013) and OS (*P* = 0.004), and high cytoplasmic S100A10 staining was significantly associated with reduced OS (*P* = 0.027)^20^. However, they did not evaluate its predictive performance of ovarian cancer prognosis and only the specific protein was studied. Although recent large-scale genomic, proteomic and metabolomic analyses have been performed to identify the potential biomarkers in the OS prediction across various cancers^21–23^, these biomarkers are not widely used in clinical practice. One of the main reasons is that the statistical methods that are used for biomarker selection do not consider the interaction between proteins or genes, which lead to unduplicated biomarkers. In this study, the SOGL method can combine priori biological knowledge to select the potential biomarkers. These results are in line with the reality of biological relationship.

Metabolic pathways have been reported to play an important role in the diagnosis, progression and prognosis across various cancers^24–27^. Our current study also confirmed that dysregulated metabolic pathway has strong association with the prognosis of ovarian cancer, which consists of carbon metabolism, nicotinate and nicotinamide metabolism, TCA cycle, Alanine, aspartate and glutamate metabolism, and biosynthesis of amino acids. Among all the sub-pathways, biosynthesis of amino acids pathway achieves the best predictive performance compared with other sub-pathways in prognosis prediction of ovarian cancer. As known, amino acids often provide energy to support the proliferation of cancer cells and elevate levels of amino acids and their products are pathogenic factors for oxidative stress, neurological disorders and cancers. The tumor cells universally displayed high accumulation of amino acid^28^. Galactose metabolism together with fructose and mannose metabolism, from amino sugar and nucleotide sugar metabolism, also have relationship with ovarian cancer survival. Cramer *et al*. demonstrated the increase in galactose consumption is positively associated with the risk of ovarian cancer based on a blood galactose metabolism^29^. Meanwhile, fructose enhances protein and nucleotide synthesis and appears to promote a more aggressive cancer phenotype^30, 31^. Several researches revealed that Carbon metabolism is tightly related to the progress of cancers, and found that the activity of ENO3 and PGAM were upregulated in tumor cells^32, 33^, which were consistent with our current study. Tricarboxylic acid (TCA) cycle points to a pivotal role of altered glucose and energy metabolism in cancers and Migita *et al*. indicated that ATP-citrate lyase (ACLY), a key enzyme for lipid synthesis, is frequently overexpressed or activated in cancer to promote lipid synthesis and tumor progression. ACLY activity was found to be significantly higher than normal lung tissue, which is also a chemical inhibitor^34–36^. Overexpression of pyruvate dehydrogenase B (PDHB) could inhibit the growth of ovarian cancer cells^37^ and researches have linked this pathway to worse prognosis in ovarian, kidney, colon and lung adenocarcinoma^38–41^. Zhang *et al*. found three pathways involved in the regulation of actin cyto-skeleton, apoptosis and adherens junction were associated with outcome of HGSC based on the enrichment of survival related proteins^12^. These three pathways also selected in our current study, but the predictive performance was not satisfactory compared with others.

Three proteins were selected as predictive proteins and highly expressed with the prolongation of the survival time. EIF2B is essential in all cells of the body for protein synthesis under different stress conditions, and there were five eukaryotic initiation factor 2B subunits encoded EIF2B namely EIF2B1, EIF2B2, EIF2B3, EIF2B4, EIF2B5, which was known to cause white matter abnormalities^42^, but no studies have been reported that EIF2B might be related with cancers. The relationship between EIF2B and cancers need further study. PRPS1L1 is the abbreviation for phosphoribosyl pyrophosphate synthetase 1-like 1 and with the function of kinase activity, lipoate-protein ligase B activity, magnesium ion binding, ribose phosphate diphosphokinase activity, transferase activity. PRPS1L1 participates the biosynthesis of amino acids pathway. Evidence shown that activity of RAS-MAPK pathway could drive cell proliferation^43^. However, Annabell *et al*. revealed that hyperactive of MAPK induced loss of ERα expression in breast cancer and tumors, which could allow for restoration of tamoxifen sensitivity. In present study, higher expression of MAPK13 and MAPK14 were correlated with a better prognosis, which led to patients more sensitive to the chemotherapy and prolong the survival time of cancer patients^44^.

In summary, we utilized a reliable and novel biomarker selection method and strategy to identify the dysregulated molecular pathways and individual molecule that associated with survival in HGSC. Biosynthesis of amino acids and a panel of three proteins are associated with the survival of HGSC patients. More importantly, a risk score, which might be transformed into clinical practice, facilitates and improves the current clinical predictors. Our study provides additional information for further mechanism research and therapeutic avenues to improve patient outcomes.

## Methods

### Patient dataset

The present analysis dataset was obtained through the CPTAC database (https://cptac-data-portal.georgetown.edu/cptac/s/S026), as described previously^12^, which consists of 9600 proteins and clinical data from 169 HGSC patients at two independent centers, Johns Hopkins University (JHU, n = 119) and Pacific Northwest National Laboratory (PNNL, n = 82). Zhang *et al*. indicated all patients in this study were newly diagnosed with ovarian serous adenocarcinoma without pretreatment and underwent surgical resection.

### Data preparation

We firstly computed the median, log2 relative protein abundance over 4,476 proteins presented in every sample and used re-centering to achieve a common median of 0 to overcome the differences in laboratory condition. 32 samples were overlapped at JHU and PNNL, which were used to correct for laboratory-related differences in the log2 relative abundances at individual protein levels between the two sites. The specific method was shifting the PNNL data at individual protein levels so that median abundances of each protein estimated over the 32 overlapping samples at PNNL and JHU were equalized and eliminated the batch effects. Proteins with missing data were excluded from the analysis to avoid problems associated with the imputation of missing values. The mRNA expression for the 169 HGSC tumors analyzed in this study was obtained from FIREHOSE (https://confluence.broadinstitute.org/display/GDAC/Home). 3,586 proteins paired proteome-genome were used to further analysis. Due to more than one protein was mapped to a gene, we selected a representative (minimum RefSeq ID) protein and reduced the number of proteins from 3,586 down to 3,329.

### Protein pathway enrichment

KEGG is a database resource integrate molecular-level information, especially large-scale molecular datasets generated by genome sequencing and other high-throughput experimental technologies. KEGG PATHWAY is a collection of manually drawn pathway maps representing our knowledge on the molecular interaction and reaction networks^45, 46^. To group the proteins into different pathways based on the biological function, r packages “org.Hs.eg.db^47^” and “clusterProfile^48^” were utilized to identify the corresponding KEGG-IDs of proteins and performed KEGG enrichment analysis for them, respectively. Enrichment pathways analysis that fdr value < 0.05 were selected for further study.

### Identification of survival related biological pathways and crucial proteins

Since one protein may map to multiple biological pathways in the process of KEGG enrichment analysis and SOGL^49^ is effective for sparse linear predictors in both predefined groups and within groups, especially for the condition that overlapping features in different groups. We took SOGL method to identify the main pathways and crucial proteins related to the OS of ovarian cancer. Coefficients of proteins had effect on the outcomes were estimated to nonzero, when the coefficients < 0 mean highly expressed with the prolongation of the survival time and coefficients > 0 mean lowly expressed with the prolongation of the survival time. Pathways of selected proteins were regarded as dysregulation pathways related to survival. Here the groups were defined as the biological pathways and within groups’ features were defined as proteins in each pathway based on the KEGG enrichment analysis.

### Relative importance of pathways in predicting OS

In order to compare the pathway importance to the OS among the selected pathways, a relative pathway score was defined as a linear combination of proteins in each pathway and coefficient for each protein was weighted by their respective sparse linear coefficient. Time-dependent area under the receiver operating characteristic (ROC) curve^50^, allowing characterization of diagnostic accuracy for censored survival outcomes, was explored to evaluate the predictive accuracy of survival based on each pathway score. Plug-in MetScape app for Cytoscape was utilized to visualize the relationship between selected proteins and corresponding compounds biologically.

### Protein-driven risk score and its predictive performance

In order to facilitate the clinical application, we selected predictive proteins based on univariate AUC > 0.90 and CC < 0.30 between each pair of proteins, which indicated that these predictive proteins had high predictive accuracy but relative independence. We further constructed a protein-driven risk score, a linear combination of predictive proteins, coefficient for each protein in the predictive risk score was weighted by their respective Cox regression coefficients. Cox proportional hazards model was utilized to analysis whether protein-driven risk scores were independent of clinical predictors for HGSC survival including univariate and multivariate analysis. Time-dependent AUC was explored to evaluate the predictive performance of protein-driven risk scores alone and protein-driven risk scores together with clinical factors in OS and PFS. In order to visualize the relationship between predictive risk scores and survival time (OS & PFS) clearly, we categorized predictive risk score into low, medium, and high risk groups, based on its corresponding 25^th^ and the 75^th^ percentiles as cutoffs. Survival curves of the risk score were calculated by the Kaplan-Meier method and compared using the log-rank test among groups.

### Sparse overlapping group lasso

Sparse overlapping group lasso method aiming to solve the problem that duplicated variables in different groups. SOGL is derived from the lasso^51^ and group lasso method^52^. The coefficients of variables are as follows:

1 $$\hat{\beta }=\text{arg}\mathop{\min }\limits_{\beta }\frac{1}{n}[\mathrm{log}(\sum _{i\in D}(\sum _{j\in {R}_{i}}\exp ({\tilde{X}}_{j}\tilde{v})-{\tilde{X}}_{i}\tilde{v}))]+\lambda \{(1-\alpha )\sum _{g\in \varsigma }{d}_{g}{\Vert {\tilde{v}}^{g}\Vert }_{2}+\alpha {\Vert \tilde{v}\Vert }_{1}\}$$

where ${\Vert \cdot \Vert }_{1}$ and ${\Vert \cdot \Vert }_{2}$ is the Euclidean norm, $\tilde{X}$ is an $n\times ({\sum }_{g\in \varsigma }|g|)$-duplicated matrix. $\tilde{v}$ is a $n\times ({\sum }_{g\in \varsigma }|g|)$-dimensional vector. ${d}_{g}$ is a positive weight (i.e., the size of the ${g}^{th}$ group), α ∈ [0,1] -a convex combination of the lasso and group lasso penalties, $\lambda$ used to adjust the sparsity of the solution, ${\tilde{v}}^{g}$ is a group latent variables^53^, *D* is the set of failure indices, ${R}_{{\rm{i}}}$ is the set of indices, j, with ${{\rm{y}}}_{j}\ge {y}_{i}$ (those still at risk at failure time *i*). In the actual data analysis, *X* are the submatrices by group, *Y* corresponding to failure/censoring times and the status for each observation (failure/censoring). This method can perform not only group selection but variable selection within the selected groups, especially in the condition that duplicated variables in different groups. There were two parameters, λ and α, in the model of SOGL. We fixed the mixing parameter α and computed solutions for a path of λ values (as λ regulates the degree of sparsity). The values of λ was sufficiently large to set $\hat{\beta }$ = 0, and decrease λ until we are near the unregularized solution. In current study, we expected strong group-wise sparsity and we have used α = 0.05. The model was fit for a path of 20 λ-values with λ_min_ = 0.1λ_max_. The final value of λ was set to the value where the minimum value of negative log likelihoods of the model by 10-fold cross-validation^54^.

### Time-dependent AUC

Time-dependent AUC was utilized to evaluate the predictive performance of a continuous diagnostic marker, *X*, with the outcomes are time dependent, *D(t)*. If a patient has died prior to time *t*, *D(t)* = 1and zero otherwise. Heagerty *et al*. proposed summarizing the discrimination potential of a marker *X*, measured at baseline (*t* = 0), by calculating ROC curves for cumulative disease or death incidence by time *t*, which we denote as ROC(*t*)^19^. The time-dependent sensitivity and specificity functions are defined as:

2 $${\rm{sensitivity}}(c,t)=P\{X > c|D(t)=1\}$$

3 $${\rm{specificity}}(c,t)=P\{X\le c|D(t)=0\}$$

The corresponding ROC(*t*) curve for any time *t* is defined as the plot of {sensitivity(*c*, *t*)} versus {specificity(*c*, *t*)}, with cutoff point *c* varying^55^. In the current study, a 10-fold cross-validation was used to evaluate the predictive performance.

## Electronic supplementary material

Supplementary metarials

Dataset 1
