# Supplementary material for: Proteomics analysis to reveal biological pathways and predictive proteins in the survival of high-grade serous ovarian cancer
Source: Sci Rep. 2017 Aug 29;7:9896. doi: 10.1038/s41598-017-10559-9 (PMC5575023; doi:10.1038/s41598-017-10559-9)
Supplement: Supplementary file 1 — Supplementary metarials [file 41598_2017_10559_MOESM1_ESM.pdf]

Proteomics analysis to reveal biological pathways and predictive proteins in the survival of high-grade serous ovarian cancer

Hongyu Xie[1, †], Wenjie Wang[1, †], Fengyu Sun[2], Kui Deng[1], Xin Lu[3], Huijuan Liu[1], Weiwei Zhao[1], Yuanyuan Zhang[1], Xiaohua Zhou[4], Kang Li[1,*], Yan Hou[1,*]

Supplementary Table S1. 75 Significant pathways of enrichment analysis based on fdr < 0.05

Num	Pathway	fdr	Count
1	Carbon metabolism	6.01E-28	86
2	Metabolic pathways	7.22E-21	450
3	Valine, leucine and isoleucine degradation	1.20E-15	40
4	Glycolysis / Gluconeogenesis	3.56E-14	48
5	Citrate cycle (TCA cycle)	9.32E-14	28
6	Biosynthesis of amino acids	5.96E-13	50
7	Pathogenic Escherichia coli infection	2.42E-12	40
8	Spliceosome	1.04E-11	72
9	Pyruvate metabolism	2.28E-10	30
10	Propanoate metabolism	5.51E-10	26
11	Fatty acid degradation	3.70E-09	31
12	Focal adhesion	3.70E-09	91
13	Protein processing in endoplasmic reticulum	1.24E-08	77
14	Complement and coagulation cascades	1.27E-08	45
15	Amino sugar and nucleotide sugar metabolism	1.31E-08	32
16	Parkinson's disease	1.87E-08	68
17	Glutathione metabolism	3.34E-08	34
18	Oxidative phosphorylation	3.96E-08	64
19	RNA transport	2.88E-07	75
20	Shigellosis	2.88E-07	37
21	Huntington's disease	3.43E-07	82
22	Alzheimer's disease	6.16E-07	74
23	Cysteine and methionine metabolism	9.07E-07	28
24	Regulation of actin cytoskeleton	9.89E-07	88
25	Pentose phosphate pathway	1.78E-06	21
26	Prion diseases	2.68E-06	23
27	Endocytosis	3.42E-06	100
28	Platelet activation	4.83E-06	55
29	Bacterial invasion of epithelial cells	8.10E-06	39
30	Phagosome	8.10E-06	65
31	Proteasome	8.10E-06	26
32	Glyoxylate and dicarboxylate metabolism	1.12E-05	19
33	Lysosome	8.15E-05	52

34	ECM-receptor interaction	9.55E-05	38
35	Leukocyte transendothelial migration	0.000104	50
36	Endocrine and other factor-regulated calcium reabsorption	0.000142	25
37	2-Oxocarboxylic acid metabolism	0.000161	13
38	Fructose and mannose metabolism	0.0003	19
39	Tight junction	0.000372	55
40	beta-Alanine metabolism	0.000384	18
41	Arginine and proline metabolism	0.000479	25
42	Amoebiasis	0.000505	42
43	Vibrio cholerae infection	0.000674	25
44	Salmonella infection	0.000674	37
45	Proteoglycans in cancer	0.000727	74
46	Alanine, aspartate and glutamate metabolism	0.000727	19
47	Aminoacyl-tRNA biosynthesis	0.00081	30
48	Protein export	0.001007	14
49	Proximal tubule bicarbonate reclamation	0.001007	14
50	Galactose metabolism	0.001253	17
51	Adherens junction	0.001426	32
52	Fatty acid metabolism	0.001579	23
53	Fc gamma R-mediated phagocytosis	0.001627	38
54	Non-alcoholic fatty liver disease (NAFLD)	0.001701	56
55	mRNA surveillance pathway	0.002076	37
56	Pertussis	0.002295	32
57	Central carbon metabolism in cancer	0.002323	29
58	Epithelial cell signaling in Helicobacter pylori infection	0.003052	29
59	DNA replication	0.003104	18
60	Butanoate metabolism	0.003257	15
61	HIF-1 signaling pathway	0.00338	40
62	Mismatch repair	0.003549	13
63	Gap junction	0.004012	35
64	Insulin signaling pathway	0.004012	51
65	Tryptophan metabolism	0.004502	19
66	Nicotinate and nicotinamide metabolism	0.00474	15
67	Legionellosis	0.011736	23
68	Apoptosis	0.011903	49
69	Collecting duct acid secretion	0.020225	13
70	Glucagon signaling pathway	0.020635	37
71	Renal cell carcinoma	0.020855	26
72	Starch and sucrose metabolism	0.021097	16
73	Arrhythmogenic right ventricular cardiomyopathy (ARVC)	0.022762	28
74	Terpenoid backbone biosynthesis	0.024488	11
75	Epstein-Barr virus infection	0.032639	65

Abbreviations: false discovery rate (fdr); The number of proteins in each selected pathway (Count).

Supplementary Table S2. 50 pathways and 455 Significant proteins based on Sparse overlapping group lasso analysis

Num	Protein ID	Protein Name	Gene ID	Beta	*P* value	Protein AUC	Pathway	Pathway AUC
1	NP_000017	ADSL	158	0.1397	0.4776	0.508	Metabolic pathways	0.881
2	NP_000039	ASL	435	4.8799	0.0264	0.762		
3	NP_000047	BCKDHB	594	-0.4113	0.2332	0.564		
4	NP_000134	FH	2271	-5.9995	0.7604	0.556		
5	NP_000145	GALK1	2584	-4.8122	0.2893	0.608		
6	NP_000243	MTM1	4534	3.5201	0.7581	0.506		
7	NP_000246	MUT	4594	-3.1981	0.0438	0.673		
8	NP_000281	PGAM2	5224	5.9050	0.0064	0.628		
9	NP_000294	PMM2	5373	-3.8718	0.0763	0.534		
10	NP_000365	UROD	7389	-1.1085	0.3071	0.527		
11	NP_000437	PON1	5444	-3.8859	0.6061	0.784		
12	NP_000469	ALPL	249	1.7712	0.4333	0.636		
13	NP_000476	APRT	353	6.5637	0.0660	0.677		
14	NP_000503	GALNS	2588	0.2721	0.6995	0.526		
15	NP_000659	ADH1B	125	1.0054	0.0939	0.62		
16	NP_000660	ADH1C	126	4.3874	0.1244	0.629		
17	NP_000681	ALDH2	217	-2.2465	0.7337	0.575		
18	NP_000683	ALDH1B1	219	-1.1174	0.5325	0.641		
19	NP_000687	ALDH9A1	223	-0.3857	0.0881	0.667		
20	NP_000700	BCKDHA	593	1.7205	0.6271	0.577		
21	NP_000779	DCK	1633	-1.5181	0.1696	0.538		
22	NP_000916	PDHB	5162	0.8072	0.5765	0.518		

23	NP_000923	PLCB3	5331	3.7758	0.3346	0.742
24	NP_001024	RRM1	6240	-0.2617	0.0672	0.659
25	NP_001032238	ACOT1	641371	-0.2611	0.8852	0.562
26	NP_001087	ACLY	47	5.8300	0.0275	0.626
27	NP_001123237	NSDHL	50814	-1.6627	0.1306	0.554
28	NP_001157412	PYGL	5836	4.8024	0.0726	0.574
29	NP_001165948	RRM2B	50484	3.7836	0.2122	0.5
30	NP_001766	CD38	952	-2.5271	0.0447	0.668
31	NP_001853	COX5B	1329	0.4779	0.3646	0.548
32	NP_001957	EHHADH	1962	0.0498	0.9729	0.638
33	NP_002067	GNS	2799	0.2059	0.5426	0.573
34	NP_002331	LSS	4047	3.0238	0.2137	0.687
35	NP_002504	NME3	4832	-0.6288	0.4764	0.582
36	NP_002517	NT5E	4907	0.3520	0.0026	0.686
37	NP_002617	PFKL	5211	1.6996	0.7550	0.622
38	NP_002694	PPAT	5471	-0.7064	0.0809	0.573
39	NP_003095	SORD	6652	1.3198	0.2497	0.686
40	NP_003725	AOC3	8639	0.0257	0.0201	0.611
41	NP_003841	SUCLA2	8803	3.6396	0.2719	0.557
42	NP_004068	CS	1431	1.0668	0.8150	0.647
43	NP_004453	FDFT1	2222	-0.8301	0.1334	0.682
44	NP_004472	GALNT2	2590	-6.0236	0.3014	0.518
45	NP_004484	HSD17B10	3028	-2.0842	0.1611	0.579
46	NP_004542	NDUFS3	4722	-1.5030	0.9947	0.637
47	NP_004896	PRDX6	9588	-3.6054	0.2087	0.505
48	NP_004997	NDUFS1	4719	-0.2138	0.8304	0.789

49	NP_005551	LAMA5	3911	1.1957	0.9291	0.592
50	NP_005737	NAMPT	10135	4.3900	0.1637	0.667
51	NP_006310	CDIPT	10423	3.8106	0.1093	0.683
52	NP_006406	SPTLC1	10558	5.3106	0.1100	0.506
53	NP_006547	PMVK	10654	-3.5744	0.0154	0.529
54	NP_006812	ACOT2	10965	-0.2707	0.8854	0.562
55	NP_008986	POLR3A	11128	3.4661	0.7134	0.529
56	NP_036277	DTYMK	1841	12.5354	0.0912	0.709
57	NP_036475	NNT	23530	-1.6279	0.0136	0.653
58	NP_037460	PYCR2	29920	0.0670	0.4708	0.776
59	NP_057041	APIP	51074	-1.2586	0.1227	0.502
60	NP_057392	CMPK1	51727	-11.5721	0.0202	0.783
61	NP_057452	ISYNA1	51477	-2.7163	0.4062	0.765
62	NP_057594	SCLY	51540	-2.9301	0.2177	0.623
63	NP_059119	GALNT7	51809	2.1000	0.7089	0.662
64	NP_060705	CNDP2	55748	4.2286	0.1219	0.596
65	NP_060831	AGPAT5	55326	-0.4020	0.1692	0.532
66	NP_061156	CMAS	55907	7.0337	0.1023	0.578
67	NP_061819	NANS	54187	0.9695	0.5403	0.538
68	NP_061982	ALG1	56052	2.7998	0.5765	0.506
69	NP_065207	GALNT1	2589	-0.1963	0.7054	0.561
70	NP_066552	NDUFV2	4729	-7.6737	0.5990	0.593
71	NP_068806	GMPPB	29925	-1.3038	0.0736	0.788
72	NP_077001	DCTPP1	79077	2.3779	0.6064	0.57
73	NP_078836	ACSS3	79611	1.3039	0.0869	0.578
74	NP_079406	HKDC1	80201	-1.4229	0.6534	0.58

75	NP_112485	FAHD1	81889	-0.3645	0.4449	0.592		
76	NP_443739	ENO3	2027	2.3353	0.0093	0.816		
77	NP_620156	GALM	130589	-0.7493	0.1113	0.841		
78	NP_653164	RPIA	22934	2.4517	0.9154	0.509		
79	NP_660202	NAPRT	93100	-8.1363	0.1365	0.523		
80	NP_665877	GSTZ1	2954	2.6069	0.9506	0.539		
81	NP_787082	PRPS1L1	221823	-5.0536	0.0032	0.918		
82	NP_849193	STT3B	201595	-0.0083	0.7871	0.648		
83	NP_872282	COQ6	51004	1.1867	0.3009	0.545		
84	NP_958800	FLAD1	80308	1.1929	0.8966	0.61		
85	NP_976251	ACSL3	2181	2.6306	0.1494	0.565		
86	NP_001005336	DNM1	1759	-0.3904	0.6120	0.511		
87	NP_001005751	FAM21A	387680	2.8032	0.0204	0.533		
88	NP_001155901	PDCD6IP	10015	2.7766	0.1009	0.566		
89	NP_001273	AP2B1	163	-0.2083	0.3686	0.607		
90	NP_002731	PRKCI	5584	2.8254	0.1871	0.57		
91	NP_004184	GBF1	8729	0.5662	0.6870	0.542		
92	NP_004860	VPS4B	9525	-0.5277	0.9049	0.545		
93	NP_006454	STAMBP	10617	5.6011	0.0975	0.8	Endocytosis	0.767
94	NP_055241	SNX5	27131	2.4503	0.0109	0.514		
95	NP_055902	SPG20	23111	-1.0318	0.4684	0.616		
96	NP_064647	ARRB1	408	1.8000	0.3429	0.578		
97	NP_073570	SMAP2	64744	2.0808	0.6947	0.523		
98	NP_112240	ARPC5L	81873	0.6787	0.4130	0.763		
99	NP_113671	ITCH	83737	0.5742	0.6197	0.703		
100	NP_115729	VPS25	84313	3.8900	0.2844	0.675		

101	NP_115765	ARFGAP2	84364	-1.1584	0.0686	0.588		
102	NP_644670	EHD4	30844	7.1585	0.0058	0.593		
103	NP_001074324	PXN	5829	0.0130	0.1165	0.62		
104	NP_001138360	BAIAP2	10458	0.5336	0.0746	0.756		
105	NP_001170785	RRAS2	22800	-0.6214	0.4404	0.614		
106	NP_002619	PFN2	5217	3.1836	0.4370	0.563		
107	NP_002863	RAC2	5880	0.7857	0.1516	0.571		
108	NP_002897	RDX	5962	0.6105	0.0746	0.516		
109	NP_003861	IQGAP1	8826	-0.1344	0.2945	0.657		
110	NP_003890	ARHGEF7	8874	0.9970	0.6270	0.511		
111	NP_004831	ARHGEF6	9459	0.0773	0.4008	0.716	Regulation of actin cytoskeleton	0.613
112	NP_004915	ACTN4	81	1.6283	0.0000	0.726		
113	NP_005397	ROCK1	6093	0.3164	0.0757	0.523		
114	NP_006261	RRAS	6237	-1.2217	0.0854	0.718		
115	NP_006563	GNA13	10672	-0.1342	0.7977	0.565		
116	NP_009293	DIAPH2	1730	-1.4658	0.9029	0.798		
117	NP_112240	ARPC5L	81873	0.3136	0.4130	0.763		
118	NP_722560	PTK2	5747	0.3780	0.3420	0.523		
119	NP_945353	ARHGEF1	9138	2.3560	0.1884	0.619		
120	NP_001284	CLNS1A	1207	-0.9316	0.0259	0.641		
121	NP_001403	EIF1AX	1964	-0.3857	0.0451	0.698		
122	NP_001405	EIF2B1	1967	-5.7934	0.0020	0.934		
123	NP_002902	UPF1	5976	-0.9336	0.4203	0.557	RNA transport	0.798
124	NP_003391	XPO1	7514	-1.1561	0.0069	0.65		
125	NP_003746	EIF3G	8666	-0.1660	0.2516	0.503		
126	NP_003749	EIF3J	8669	2.1873	0.1172	0.577		

127	NP_005078	FXR1	8087	0.0398	0.5888	0.69		
128	NP_005830	SRRM1	10250	-0.5168	0.1564	0.561		
129	NP_005866	EIF1B	10289	1.7687	0.3426	0.545		
130	NP_009166	XPOT	11260	4.9198	0.3746	0.754		
131	NP_060700	NUP133	55746	-3.2595	0.0705	0.574		
132	NP_065098	EIF2B3	8891	-0.4614	0.0025	0.73		
133	NP_065801	XPO5	57510	-0.9392	0.0523	0.587		
134	NP_077315	THOC6	79228	-5.9375	0.0073	0.813		
135	NP_892116	EIF5	1983	-1.1450	0.0132	0.825		
136	NP_001019820	CANX	821	1.6075	0.7559	0.654	Protein processing in endoplasmic reticulum	0.795
137	NP_001026859	PLAA	9373	-1.7782	0.0132	0.818		
138	NP_001036199	SEC24B	10427	6.2450	0.0766	0.761		
139	NP_001530	DNAJA1	3301	-2.6152	0.0920	0.716		
140	NP_002750	EIF2AK2	5610	0.8394	0.9341	0.556		
141	NP_003135	SSR1	6745	-2.2299	0.0611	0.567		
142	NP_005177	CAPN1	823	0.7473	0.0576	0.654		
143	NP_005304	PDIA3	2923	1.5972	0.1137	0.62		
144	NP_005561	LMAN1	3998	-2.0516	0.9444	0.566		
145	NP_005852	STUB1	10273	-5.0589	0.5475	0.505		
146	NP_006816	CKAP4	10970	-0.1911	0.6011	0.609		
147	NP_009057	VCP	7415	-0.7547	0.0837	0.75		
148	NP_056516	ERLEC1	27248	-4.2088	0.0461	0.647		
149	NP_064505	UGGT1	56886	-0.0561	0.2112	0.658		
150	NP_110437	TXNDC5	81567	-1.5497	0.1883	0.586		
151	NP_849193	STT3B	201595	-0.0009	0.7871	0.648		
152	NP_000160	GLA	2717	0.1344	0.6663	0.522	Lysosome	0.712

153	NP_000226	LIPA	3988	-0.2226	0.7760	0.577		
154	NP_000253	NAGA	4668	0.0683	0.3611	0.618		
155	NP_000382	TPP1	1200	-0.2013	0.4505	0.741		
156	NP_000387	CTSK	1513	0.1605	0.1844	0.661		
157	NP_000503	GALNS	2588	0.1370	0.6995	0.526		
158	NP_001601	ACP2	53	-0.7218	0.0532	0.791		
159	NP_001900	CTSD	1509	-0.7828	0.5674	0.593		
160	NP_002067	GNS	2799	0.1017	0.5426	0.573		
161	NP_002950	SORT1	6272	-2.1921	0.0188	0.563		
162	NP_003929	AP3D1	8943	-1.3596	0.5880	0.539		
163	NP_005597	LGMN	5641	2.2361	0.2756	0.514		
164	NP_006423	NPC2	10577	-1.3798	0.4704	0.629		
165	NP_036227	AP3M1	26985	-1.1053	0.3377	0.595		
166	NP_000066	CDK4	1019	-1.7428	0.2169	0.672		
167	NP_001123910	SPTAN1	6709	-0.6812	0.8193	0.552		
168	NP_001159477	EPB41	2035	0.0545	0.3474	0.639		
169	NP_001170785	RRAS2	22800	-0.2595	0.4404	0.614		
170	NP_001617	AKT2	208	1.0577	0.2298	0.64		
171	NP_002061	GNAI2	2771	-0.1248	0.6462	0.511		
172	NP_002731	PRKCI	5584	0.7659	0.1871	0.57	Tight junction	0.589
173	NP_004380	CTNNA2	1496	-0.7632	0.1960	0.611		
174	NP_004915	ACTN4	81	1.1633	0.0000	0.726		
175	NP_005326	HCLS1	3059	0.0093	0.3839	0.633		
176	NP_006261	RRAS	6237	-0.8880	0.0854	0.718		
177	NP_006487	GNAI3	2773	-0.6990	0.7439	0.582		
178	NP_997704	PRKCD	5580	0.5517	0.6210	0.842		

179	NP_000923	PLCB3	5331	0.4853	0.3346	0.742		
180	NP_001142	SLC25A4	291	0.2912	0.5278	0.613		
181	NP_001143	SLC25A5	292	0.3891	0.4818	0.614		
182	NP_001177765	DCTN1	1639	0.3883	0.0454	0.618		
183	NP_001273	AP2B1	163	-0.0137	0.3686	0.607		
184	NP_001627	SLC25A6	293	0.3183	0.5343	0.594		
185	NP_001853	COX5B	1329	0.0975	0.3646	0.548	Huntington's disease	0.584
186	NP_003192	TFAM	7019	-1.7750	0.2306	0.556		
187	NP_004542	NDUFS3	4722	-0.3174	0.9947	0.637		
188	NP_004997	NDUFS1	4719	-0.0709	0.8304	0.789		
189	NP_005329	HIP1	3092	0.6076	0.3028	0.696		
190	NP_009034	NDUFV1	4723	-0.0519	0.4715	0.751		
191	NP_066552	NDUFV2	4729	-1.1450	0.5990	0.593		
192	NP_000134	FH	2271	-0.0776	0.7604	0.556		
193	NP_000246	MUT	4594	-0.0367	0.0438	0.673		
194	NP_000281	PGAM2	5224	0.0715	0.0064	0.628		
195	NP_000916	PDHB	5162	0.0077	0.5765	0.518		
196	NP_001957	EHHADH	1962	0.0040	0.9729	0.638		
197	NP_002617	PFKL	5211	0.0292	0.7550	0.622	Carbon metabolism	0.593
198	NP_003841	SUCLA2	8803	0.0510	0.2719	0.557		
199	NP_004068	CS	1431	0.0063	0.8150	0.647		
200	NP_079406	HKDC1	80201	-0.0182	0.6534	0.58		
201	NP_443739	ENO3	2027	0.0377	0.0093	0.816		
202	NP_653164	RPIA	22934	0.0288	0.9154	0.509		
203	NP_787082	PRPS1L1	221823	-0.0661	0.0032	0.918		
204	NP_000281	PGAM2	5224	0.0142	0.0064	0.628	Central carbon metabolism in cancer	0.593

205	NP_000916	PDHB	5162	0.0010	0.5765	0.518		
206	NP_001617	AKT2	208	0.0123	0.2298	0.64		
207	NP_002617	PFKL	5211	0.0055	0.7550	0.622		
208	NP_004198	SLC16A3	9123	0.0043	0.0843	0.568		
209	NP_079406	HKDC1	80201	-0.0037	0.6534	0.58		
210	NP_001070962	NFKB2	4791	0.2693	0.0054	0.619		
211	NP_001167638	SYK	6850	-1.0381	0.0478	0.644		
212	NP_001617	AKT2	208	0.5754	0.2298	0.64		
213	NP_001766	CD38	952	-0.4859	0.0447	0.668		
214	NP_001767	ENTPD1	953	0.9053	0.0776	0.685		
215	NP_002745	MAPK13	5603	-0.2159	0.0124	0.914	Epstein-Barr virus infection	0.762
216	NP_002750	EIF2AK2	5610	0.0912	0.9341	0.556		
217	NP_003141	STAT3	6774	0.2550	0.1783	0.537		
218	NP_003391	XPO1	7514	-0.2196	0.0069	0.65		
219	NP_003461	USP7	7874	1.1903	0.8703	0.511		
220	NP_008986	POLR3A	11128	0.4648	0.7134	0.529		
221	NP_001074324	PXN	5829	0.0005	0.1165	0.62		
222	NP_001170785	RRAS2	22800	-0.0011	0.4404	0.614		
223	NP_001617	AKT2	208	0.0035	0.2298	0.64		
224	NP_002745	MAPK13	5603	-0.0005	0.0124	0.914		
225	NP_002897	RDX	5962	0.0031	0.0746	0.516		
226	NP_003141	STAT3	6774	0.0008	0.1783	0.537	Proteoglycans in cancer	0.682
227	NP_005326	HCLS1	3059	0.0001	0.3839	0.633		
228	NP_005397	ROCK1	6093	0.0007	0.0757	0.523		
229	NP_006261	RRAS	6237	-0.0031	0.0854	0.718		
230	NP_722560	PTK2	5747	0.0010	0.3420	0.523		

231	NP_945353	ARHGEF1	9138	0.0059	0.1884	0.619		
232	NP_000923	PLCB3	5331	0.3985	0.3346	0.742		
233	NP_001167638	SYK	6850	-0.9448	0.0478	0.644		
234	NP_001617	AKT2	208	0.6688	0.2298	0.64		
235	NP_002061	GNAI2	2771	-0.1366	0.6462	0.511		
236	NP_002731	PRKCI	5584	0.4030	0.1871	0.57		
237	NP_002745	MAPK13	5603	-0.2218	0.0124	0.914	Platelet activation	0.502
238	NP_003752	VAMP8	8673	0.3353	0.9899	0.532		
239	NP_005397	ROCK1	6093	0.1329	0.0757	0.523		
240	NP_006487	GNAI3	2773	-0.4411	0.7439	0.582		
241	NP_006563	GNA13	10672	-0.0425	0.7977	0.565		
242	NP_945353	ARHGEF1	9138	0.8685	0.1884	0.619		
243	NP_001157412	PYGL	5836	2.7106	0.0726	0.574		
244	NP_001617	AKT2	208	3.0313	0.2298	0.64		
245	NP_002725	PRKAR1A	5573	0.9032	0.5533	0.623		
246	NP_002731	PRKCI	5584	2.1080	0.1871	0.57		
247	NP_002818	PTPN1	5770	-1.9610	0.4852	0.517	Insulin signaling pathway	0.633
248	NP_004231	TRIP10	9322	-7.9175	0.7178	0.589		
249	NP_004466	FLOT2	2319	3.9054	0.0605	0.632		
250	NP_005605	RHEB	6009	-3.4543	0.0050	0.869		
251	NP_006242	PRKAA1	5562	-0.3615	0.4548	0.735		
252	NP_079406	HKDC1	80201	-0.8428	0.6534	0.58		
253	NP_001074324	PXN	5829	0.0126	0.1165	0.62		
254	NP_001078930	CTNND1	1500	-3.4225	0.4140	0.528	Leukocyte transendothelial migration	0.687
255	NP_002061	GNAI2	2771	-0.1983	0.6462	0.511		
256	NP_002745	MAPK13	5603	-0.4415	0.0124	0.914		

257	NP_002863	RAC2	5880	0.5841	0.1516	0.571		
258	NP_004380	CTNNA2	1496	-0.9825	0.1960	0.611		
259	NP_004915	ACTN4	81	1.4629	0.0000	0.726		
260	NP_005397	ROCK1	6093	0.2000	0.0757	0.523		
261	NP_006487	GNAI3	2773	-0.8912	0.7439	0.582		
262	NP_722560	PTK2	5747	0.3214	0.3420	0.523		
263	NP_001020374	U2AF1	7307	-0.5785	0.1854	0.666	Spliceosome	0.573
264	NP_001035095	TCERG1	10915	-1.9509	0.3719	0.514		
265	NP_001073884	U2SURP	23350	6.5689	0.7869	0.655		
266	NP_001139019	RBM17	84991	6.9537	0.4398	0.616		
267	NP_004688	PRPF4	9128	-5.4007	0.0077	0.765		
268	NP_037425	TRA2A	29896	2.6516	0.6760	0.567		
269	NP_054722	DHX38	9785	-2.0052	0.2029	0.589		
270	NP_057131	SF3B6	51639	0.7263	0.3979	0.613		
271	NP_060362	PRPF40A	55660	-0.2146	0.0215	0.733		
272	NP_110517	CTNNBL1	56259	3.8749	0.3085	0.587		
273	NP_000387	CTSK	1513	0.2152	0.1844	0.661	Apoptosis	0.625
274	NP_001123910	SPTAN1	6709	-0.9614	0.8193	0.552		
275	NP_001217	CASP6	839	1.7807	0.8124	0.511		
276	NP_001341	DAXX	1616	2.2073	0.3992	0.809		
277	NP_001617	AKT2	208	1.3724	0.2298	0.64		
278	NP_001900	CTSD	1509	-0.8626	0.5674	0.593		
279	NP_004392	DFFA	1676	2.0753	0.0382	0.75		
280	NP_005177	CAPN1	823	0.5226	0.0576	0.654		
281	NP_061816	TUBA8	51807	-2.8115	0.1173	0.736		
282	NP_000923	PLCB3	5331	0.5026	0.3346	0.742	Alzheimer's disease	0.816

283	NP_001853	COX5B	1329	0.0451	0.3646	0.548		
284	NP_004484	HSD17B10	3028	-0.2508	0.1611	0.579		
285	NP_004542	NDUFS3	4722	-0.2413	0.9947	0.637		
286	NP_004926	CDK5	1020	-1.8995	0.0032	0.826		
287	NP_004996	NDUFB9	4715	0.0037	0.0263	0.754		
288	NP_004997	NDUFS1	4719	-0.0226	0.8304	0.789		
289	NP_005177	CAPN1	823	0.1836	0.0576	0.654		
290	NP_066552	NDUFV2	4729	-1.1745	0.5990	0.593		
291	NP_000552	GSTM1	2944	-1.5569	0.2552	0.724		
292	NP_000837	GSTA2	2939	1.3582	0.2955	0.548		
293	NP_000841	GSTM4	2948	-0.3497	0.4808	0.747		
294	NP_000842	GSTM5	2949	-4.0935	0.2321	0.61		
295	NP_001165948	RRM2B	50484	1.4442	0.2122	0.5	Glutathione metabolism	0.652
296	NP_004823	GSTO1	9446	2.5574	0.0078	0.769		
297	NP_056997	TXNDC12	51060	0.0480	0.9647	0.784		
298	NP_060040	OPLAH	26873	0.7438	0.0594	0.85		
299	NP_665683	GSTA1	2938	1.2702	0.2921	0.553		
300	NP_000047	BCKDHB	594	-0.0133	0.2332	0.564		
301	NP_000246	MUT	4594	-0.1501	0.0438	0.673		
302	NP_000427	OXCT1	5019	-0.6933	0.1174	0.556		
303	NP_000681	ALDH2	217	-0.1040	0.7337	0.575		
304	NP_000683	ALDH1B1	219	-0.0465	0.5325	0.641	Valine, leucine and isoleucine degradation	0.607
305	NP_000687	ALDH9A1	223	-0.0306	0.0881	0.667		
306	NP_000700	BCKDHA	593	0.0916	0.6271	0.577		
307	NP_001957	EHHADH	1962	0.0073	0.9729	0.638		
308	NP_004484	HSD17B10	3028	-0.1009	0.1611	0.579		

309	NP_000281	PGAM2	5224	0.2357	0.0064	0.628	Glucagon signaling pathway	0.573
310	NP_000916	PDHB	5162	0.0230	0.5765	0.518		
311	NP_000923	PLCB3	5331	0.1509	0.3346	0.742		
312	NP_001157412	PYGL	5836	0.2074	0.0726	0.574		
313	NP_001617	AKT2	208	0.1990	0.2298	0.64		
314	NP_001867	CPT1A	1374	0.2760	0.4484	0.639		
315	NP_002617	PFKL	5211	0.0840	0.7550	0.622		
316	NP_006242	PRKAA1	5562	-0.0310	0.4548	0.735		
317	NP_001014437	CARS	833	-2.5634	0.4980	0.522	Aminoacyl-tRNA biosynthesis	0.652
318	NP_001035526	YARS2	51067	1.4976	0.9213	0.594		
319	NP_002038	GARS	2617	-2.3330	0.0688	0.705		
320	NP_004530	NARS	4677	-0.1151	0.6620	0.611		
321	NP_006504	SARS	6301	-2.5331	0.0353	0.627		
322	NP_036340	HARS2	23438	0.2185	0.3761	0.832		
323	NP_060297	SARS2	54938	1.2936	0.0003	0.665		
324	NP_776049	WARS	7453	-1.5204	0.1108	0.662		
325	NP_000659	ADH1B	125	0.0069	0.0939	0.62	Fatty acid degradation	0.519
326	NP_000660	ADH1C	126	0.0349	0.1244	0.629		
327	NP_000681	ALDH2	217	-0.0144	0.7337	0.575		
328	NP_000683	ALDH1B1	219	-0.0073	0.5325	0.641		
329	NP_000687	ALDH9A1	223	-0.0038	0.0881	0.667		
330	NP_001867	CPT1A	1374	0.0520	0.4484	0.639		
331	NP_001957	EHHADH	1962	0.0021	0.9729	0.638		
332	NP_976251	ACSL3	2181	0.0175	0.1494	0.565		
333	NP_001017915	INPP5D	3635	-0.1130	0.0965	0.651	Fc gamma R-mediated phagocytosis	0.514
334	NP_001167638	SYK	6850	-1.7865	0.0478	0.644		

335	NP_001617	AKT2	208	1.1393	0.2298	0.64		
336	NP_002863	RAC2	5880	0.6325	0.1516	0.571		
337	NP_004937	DOCK2	1794	-1.8535	0.5320	0.562		
338	NP_112240	ARPC5L	81873	0.2434	0.4130	0.763		
339	NP_997704	PRKCD	5580	0.6395	0.6210	0.842		
340	NP_001078930	CTNND1	1500	-5.2608	0.4140	0.528		
341	NP_001138360	BAIAP2	10458	0.9631	0.0746	0.756		
342	NP_002818	PTPN1	5770	-1.5851	0.4852	0.517		
343	NP_002863	RAC2	5880	1.1239	0.1516	0.571	Adherens junction	0.673
344	NP_003861	IQGAP1	8826	-0.0225	0.2945	0.657		
345	NP_004380	CTNNA2	1496	-1.3927	0.1960	0.611		
346	NP_004915	ACTN4	81	2.3448	0.0000	0.726		
347	NP_001005336	DNM1	1759	-0.0063	0.6120	0.511		
348	NP_001074324	PXN	5829	0.0104	0.1165	0.62		
349	NP_004380	CTNNA2	1496	-0.1687	0.1960	0.611		
350	NP_005326	HCLS1	3059	0.0065	0.3839	0.633	Bacterial invasion of epithelial cells	0.567
351	NP_036252	CD2AP	23607	0.3757	0.5893	0.634		
352	NP_112240	ARPC5L	81873	0.0626	0.4130	0.763		
353	NP_722560	PTK2	5747	0.0550	0.3420	0.523		
354	NP_000047	BCKDHB	594	-0.0202	0.2332	0.564		
355	NP_000246	MUT	4594	-0.6847	0.0438	0.673		
356	NP_000700	BCKDHA	593	0.2997	0.6271	0.577		
357	NP_001132982	ECHDC1	55862	-3.0743	0.9192	0.732	Propanoate metabolism	0.655
358	NP_001957	EHHADH	1962	0.0125	0.9729	0.638		
359	NP_003841	SUCLA2	8803	0.6109	0.2719	0.557		
360	NP_078836	ACSS3	79611	0.2706	0.0869	0.578		

361	NP_000215	KRT18	3875	4.8069	0.0620	0.574		
362	NP_001004720	NCK2	8440	-5.0220	0.4670	0.549		
363	NP_004714	ARHGEF2	9181	-0.3605	0.1215	0.59		
364	NP_005326	HCLS1	3059	0.2842	0.3839	0.633	Pathogenic Escherichia coli infection	0.65
365	NP_005397	ROCK1	6093	0.4812	0.0757	0.523		
366	NP_061816	TUBA8	51807	-4.8222	0.1173	0.736		
367	NP_112240	ARPC5L	81873	0.9004	0.4130	0.763		
368	NP_000039	ASL	435	0.3362	0.0264	0.762		
369	NP_000281	PGAM2	5224	0.3845	0.0064	0.628		
370	NP_002617	PFKL	5211	0.1170	0.7550	0.622		
371	NP_004068	CS	1431	0.0197	0.8150	0.647	Biosynthesis of amino acids	0.9
372	NP_443739	ENO3	2027	0.2031	0.0093	0.816		
373	NP_653164	RPIA	22934	0.1428	0.9154	0.509		
374	NP_787082	PRPS1L1	221823	-0.3695	0.0032	0.918		
375	NP_001002010	NT5C3A	51251	-0.2549	0.4947	0.673		
376	NP_001766	CD38	952	-2.6992	0.0447	0.668		
377	NP_002517	NT5E	4907	0.5780	0.0026	0.686		
378	NP_005737	NAMPT	10135	1.9550	0.1637	0.667	Nicotinate and nicotinamide metabolism	0.773
379	NP_036475	NNT	23530	-1.5140	0.0136	0.653		
380	NP_660202	NAPRT	93100	-3.4408	0.1365	0.523		
381	NP_001136037	CPSF7	79869	-1.2417	0.0078	0.798		
382	NP_002902	UPF1	5976	-0.2758	0.4203	0.557		
383	NP_059133	CPSF2	53981	-0.8944	0.0187	0.653		
384	NP_060564	GSPT2	23708	0.7546	0.5273	0.508	mRNA surveillance pathway	0.768
385	NP_079498	WDR82	80335	-0.6491	0.1889	0.594		
386	NP_733839	MSI2	124540	0.1014	0.7415	0.53		

387	NP_000145	GALK1	2584	-0.2551	0.2893	0.608	Amino sugar and nucleotide sugar metabolism	0.615
388	NP_000294	PMM2	5373	-0.2053	0.0763	0.534		
389	NP_061156	CMAS	55907	0.4283	0.1023	0.578		
390	NP_061819	NANS	54187	0.0562	0.5403	0.538		
391	NP_068806	GMPPB	29925	-0.0646	0.0736	0.788		
392	NP_079406	HKDC1	80201	-0.0879	0.6534	0.58		
393	NP_000055	C3	718	-2.0214	0.9862	0.889	Legionellosis	0.748
394	NP_001070962	NFKB2	4791	0.5365	0.0054	0.619		
395	NP_001395	EEF1G	1937	-0.8237	0.0087	0.774		
396	NP_009057	VCP	7415	-0.4846	0.0837	0.75		
397	NP_056182	BCL2L13	23786	-0.8279	0.3352	0.582		
398	NP_002583	PCNA	5111	0.2650	0.1609	0.772	DNA replication	0.827
399	NP_002906	RFC3	5983	-0.7496	0.0195	0.774		
400	NP_002936	RPA1	6117	-2.6874	0.0360	0.739		
401	NP_002938	RPA3	6119	-0.2122	0.4929	0.593		
402	NP_004102	FEN1	2237	-1.9249	0.0018	0.86		
403	NP_002619	PFN2	5217	1.7205	0.4370	0.563	Shigellosis	0.57
404	NP_002745	MAPK13	5603	-0.1169	0.0124	0.914		
405	NP_005326	HCLS1	3059	0.0100	0.3839	0.633		
406	NP_005397	ROCK1	6093	0.0959	0.0757	0.523		
407	NP_112240	ARPC5L	81873	0.1588	0.4130	0.763		
408	NP_000055	C3	718	-3.2741	0.9862	0.889	Complement and coagulation cascades	0.662
409	NP_000124	F9	2158	2.9515	0.4473	0.583		
410	NP_000177	CFH	3075	-0.1052	0.5357	0.875		
411	NP_001120699	CD59	966	-6.0578	0.2811	0.641		
412	NP_001701	CFB	629	-0.2213	0.9710	0.872		

413	NP_000134	FH	2271	-0.2841	0.7604	0.556		
414	NP_000916	PDHB	5162	0.0250	0.5765	0.518		
415	NP_001087	ACLY	47	0.3011	0.0275	0.626	Citrate cycle (TCA cycle)	0.563
416	NP_003841	SUCLA2	8803	0.1818	0.2719	0.557		
417	NP_004068	CS	1431	0.0180	0.8150	0.647		
418	NP_000923	PLCB3	5331	0.1302	0.3346	0.742		
419	NP_002061	GNAI2	2771	-0.0324	0.6462	0.511	Gap junction	0.721
420	NP_006487	GNAI3	2773	-0.1160	0.7439	0.582		
421	NP_061816	TUBA8	51807	-0.3273	0.1173	0.736		
422	NP_002583	PCNA	5111	0.2047	0.1609	0.772		
423	NP_002906	RFC3	5983	-0.5477	0.0195	0.774	Mismatch repair	0.734
424	NP_002936	RPA1	6117	-2.1300	0.0360	0.739		
425	NP_002938	RPA3	6119	-0.1474	0.4929	0.593		
426	NP_000055	C3	718	-0.1887	0.9862	0.889		
427	NP_002061	GNAI2	2771	-0.0202	0.6462	0.511	Pertussis	0.829
428	NP_002745	MAPK13	5603	-0.0223	0.0124	0.914		
429	NP_006487	GNAI3	2773	-0.0628	0.7439	0.582		
430	NP_002619	PFN2	5217	1.4812	0.4370	0.563		
431	NP_002745	MAPK13	5603	-0.0984	0.0124	0.914	Salmonella infection	0.573
432	NP_005397	ROCK1	6093	0.0852	0.0757	0.523		
433	NP_112240	ARPC5L	81873	0.1436	0.4130	0.763		
434	NP_000055	C3	718	-0.1114	0.9862	0.889		
435	NP_000086	COMP	1311	-0.0147	0.2739	0.756	Phagosome	0.787
436	NP_001019820	CANX	821	0.0333	0.7559	0.654		
437	NP_061816	TUBA8	51807	-0.1069	0.1173	0.736		
438	NP_001867	CPT1A	1374	0.0124	0.4484	0.639	Fatty acid metabolism	0.632

439	NP_001957	EHHADH	1962	0.0005	0.9729	0.638		
440	NP_976251	ACSL3	2181	0.0042	0.1494	0.565		
441	NP_000039	ASL	435	1.2953	0.0264	0.762		
442	NP_002694	PPAT	5471	-0.0246	0.0809	0.573	Alanine, aspartate and glutamate metabolism	0.736
443	NP_064587	NIT2	56954	2.1833	0.0619	0.688		
444	NP_002617	PFKL	5211	0.1640	0.7550	0.622		
445	NP_653164	RPIA	22934	0.2088	0.9154	0.509	Pentose phosphate pathway	0.812
446	NP_787082	PRPS1L1	221823	-0.6143	0.0032	0.918		
447	NP_002018	FNTA	2339	-2.5397	0.2834	0.595	Terpenoid backbone biosynthesis	0.55
448	NP_006547	PMVK	10654	-2.4810	0.0154	0.529		
449	NP_004380	CTNNA2	1496	-0.1045	0.1960	0.611	Arrhythmogenic right ventricular cardiomyopathy (ARVC)	0.765
450	NP_004915	ACTN4	81	0.1409	0.0000	0.726		
451	NP_000134	FH	2271	-0.0168	0.7604	0.556	Renal cell carcinoma	0.638
452	NP_001617	AKT2	208	0.0133	0.2298	0.64		
453	NP_000427	OXCT1	5019	-1.8221	0.1174	0.556	Butanoate metabolism	0.556
454	NP_001957	EHHADH	1962	0.0127	0.9729	0.638		
455	NP_036272	SLC25A10	1468	-1.9785	0.0787	0.813	Proximal tubule bicarbonate reclamation	0.813

Supplementary Table S3. Cox univariate analysis and univariate time-dependent AUC analysis for non-overlapped proteins

Num	proteins	*P* value	Time-dependent AUC
1	EIF2B1	0.001987	0.933726
2	PRPS1L1	0.003156	0.918156
3	MAPK13	0.012446	0.913972
4	C3	0.986161	0.888841
5	CFH	0.535747	0.875223
6	CFB	0.970993	0.872337
7	RHEB	0.004952	0.869389
8	FEN1	0.00183	0.859513
9	OPLAH	0.059374	0.850155
10	PRKCD	0.621035	0.842363
11	GALM	0.111341	0.841485
12	HARS2	0.376053	0.832247
13	CDK5	0.003195	0.825767
14	EIF5	0.013205	0.824644
15	PLAA	0.013214	0.818277
16	ENO3	0.009283	0.816222
17	THOC6	0.007304	0.813283
18	SLC25A10	0.078728	0.812719
19	DAXX	0.399194	0.809484
20	STAMBP	0.097523	0.800396
21	CPSF7	0.007784	0.798212
22	DIAPH2	0.902899	0.797938
23	ACP2	0.053155	0.791469
24	NDUFS1	0.830446	0.789176
25	GMPPB	0.073602	0.787898
26	PON1	0.606145	0.783683
27	TXNDC12	0.964662	0.783643
28	CMPK1	0.020239	0.783367
29	PYCR2	0.470785	0.775828
30	EEF1G	0.008741	0.774082
31	RFC3	0.019509	0.773654
32	PCNA	0.160873	0.772208
33	GSTO1	0.007755	0.768941
34	PRPF4	0.007655	0.76542
35	ISYNA1	0.406238	0.764514
36	ARPC5L	0.413033	0.763366
37	ASL	0.026361	0.761667
38	SEC24B	0.076623	0.76138
39	COMP	0.273934	0.75641
40	BAIAP2	0.074571	0.755942
41	NDUFB9	0.026291	0.753993

42	XPOT	0.374573	0.753545
43	NDUFV1	0.471545	0.751048
44	VCP	0.083658	0.750324
45	DFFA	0.038237	0.750105
46	GSTM4	0.480771	0.746588
47	PLCB3	0.334641	0.74215
48	TPP1	0.450504	0.740932
49	RPA1	0.036013	0.739304
50	TUBA8	0.117294	0.735827
51	PRKAA1	0.454789	0.734821
52	PRPF40A	0.021478	0.732684
53	ECHDC1	0.919176	0.731664
54	EIF2B3	0.002491	0.730124
55	ACTN4	4.71E-06	0.725869
56	GSTM1	0.255189	0.723996
57	RRAS	0.085403	0.717621
58	ARHGEF6	0.400807	0.716244
59	DNAJA1	0.092006	0.71555
60	DTYMK	0.091215	0.70941
61	GARS	0.068831	0.705332
62	ITCH	0.61972	0.703325
63	EIF1AX	0.045057	0.697574
64	HIP1	0.302825	0.695552
65	FXR1	0.588839	0.690213
66	NIT2	0.061916	0.687765
67	LSS	0.213653	0.687305
68	NT5E	0.002559	0.685883
69	SORD	0.249695	0.685547
70	ENTPD1	0.077609	0.685245
71	CDIPT	0.10933	0.682578
72	FDFT1	0.133437	0.68183
73	APRT	0.065955	0.677058
74	VPS25	0.28436	0.675366
75	MUT	0.043802	0.673288
76	NT5C3A	0.494741	0.672998
77	CDK4	0.216907	0.672086
78	CD38	0.044704	0.668022
79	ALDH9A1	0.088054	0.667211
80	NAMPT	0.163673	0.666896
81	U2AF1	0.185366	0.665673
82	SARS2	0.000292	0.664629
83	WARS	0.110825	0.662424
84	GALNT7	0.708903	0.661724
85	CTSK	0.184443	0.661206

86	RRM1	0.067179	0.659031
87	UGGT1	0.211224	0.658485
88	IQGAP1	0.294519	0.656968
89	U2SURP	0.78691	0.655447
90	CAPN1	0.057612	0.654334
91	CANX	0.755865	0.654095
92	NNT	0.013553	0.652865
93	CPSF2	0.01866	0.65285
94	INPP5D	0.0965	0.650909
95	XPO1	0.006915	0.65005
96	STT3B	0.787121	0.647764
97	ERLEC1	0.046101	0.647312
98	CS	0.81498	0.646768
99	SYK	0.047796	0.644276
100	ALDH1B1	0.532482	0.641065
101	CD59	0.281102	0.64082
102	CLNS1A	0.025879	0.640694
103	AKT2	0.22978	0.640026
104	EPB41	0.347439	0.638775
105	CPT1A	0.448414	0.638771
106	EHHADH	0.972923	0.638217
107	NDUFS3	0.994664	0.637473
108	ALPL	0.433322	0.636303
109	CD2AP	0.589342	0.633732
110	HCLS1	0.38387	0.633358
111	FLOT2	0.060531	0.63187
112	NPC2	0.470374	0.629289
113	ADH1C	0.124393	0.628998
114	PGAM2	0.006356	0.627578
115	SARS	0.035336	0.627393
116	ACLY	0.027513	0.62597
117	SCLY	0.217696	0.623067
118	PRKAR1A	0.553255	0.623062
119	PFKL	0.754969	0.621633
120	ADH1B	0.093851	0.620295
121	PDIA3	0.113719	0.620279
122	PXN	0.116543	0.619582
123	ARHGEF1	0.188436	0.619187
124	NFKB2	0.005356	0.618891
125	NAGA	0.361079	0.618236
126	DCTN1	0.045415	0.617692
127	SPG20	0.468379	0.615965
128	RBM17	0.439765	0.615862
129	RRAS2	0.440411	0.613801

130	SLC25A5	0.4818	0.613547
131	SLC25A4	0.527786	0.61308
132	SF3B6	0.39794	0.612771
133	CTNNA2	0.196006	0.611408
134	AOC3	0.020091	0.610881
135	NARS	0.662004	0.610784
136	FLAD1	0.896566	0.609788
137	GSTM5	0.232052	0.609589
138	CKAP4	0.601075	0.609135
139	GALK1	0.28929	0.60845
140	AP2B1	0.368563	0.607435
141	CNDP2	0.121867	0.595712
142	AP3M1	0.337651	0.594763
143	FNTA	0.283387	0.594572
144	WDR82	0.188879	0.594118
145	YARS2	0.921314	0.593641
146	SLC25A6	0.534329	0.593514
147	RPA3	0.492912	0.593393
148	CTSD	0.567402	0.593279
149	NDUFV2	0.59895	0.59286
150	EHD4	0.005835	0.592746
151	LAMA5	0.92912	0.592202
152	FAHD1	0.444906	0.591747
153	ARHGEF2	0.121504	0.589884
154	TRIP10	0.717773	0.588811
155	DHX38	0.202873	0.588763
156	ARFGAP2	0.068568	0.588437
157	XPO5	0.052319	0.587243
158	CTNNBL1	0.308497	0.586904
159	TXNDC5	0.188321	0.58576
160	F9	0.447278	0.583458
161	NME3	0.476353	0.582238
162	GNAI3	0.743854	0.582092
163	BCL2L13	0.335198	0.581589
164	HKDC1	0.653448	0.580052
165	HSD17B10	0.161094	0.579081
166	ACSS3	0.086925	0.578406
167	ARRB1	0.342938	0.578348
168	CMAS	0.102323	0.578234
169	EIF3J	0.117198	0.577297
170	LIPA	0.776013	0.577246
171	BCKDHA	0.627113	0.576861
172	ALDH2	0.733653	0.575365
173	PYGL	0.072596	0.573986

174	NUP133	0.070533	0.573714
175	KRT18	0.062032	0.573652
176	GNS	0.542613	0.573279
177	PPAT	0.080882	0.572878
178	RAC2	0.151552	0.570827
179	PRKCI	0.187137	0.569871
180	DCTPP1	0.606354	0.569672
181	SLC16A3	0.084315	0.567651
182	SSR1	0.061071	0.567155
183	TRA2A	0.675969	0.567051
184	LMAN1	0.944409	0.565909
185	PDCD6IP	0.100906	0.565525
186	ACSL3	0.149427	0.565329
187	GNA13	0.797686	0.564703
188	BCKDHB	0.233247	0.563685
189	PFN2	0.437044	0.563243
190	SORT1	0.018824	0.56302
191	ACOT1	0.885193	0.562298
192	ACOT2	0.885414	0.562298
193	DOCK2	0.532008	0.562001
194	GALNT1	0.705424	0.560734
195	SRRM1	0.156376	0.560713
196	UPF1	0.42025	0.557455
197	SUCLA2	0.271938	0.557396
198	OXCT1	0.117394	0.555851
199	FH	0.760365	0.555786
200	TFAM	0.230561	0.555726
201	EIF2AK2	0.934121	0.555644
202	NSDHL	0.130584	0.55419
203	GSTA1	0.292085	0.553281
204	SPTAN1	0.819333	0.552464
205	NCK2	0.46703	0.548741
206	COX5B	0.364554	0.548101
207	GSTA2	0.295532	0.548031
208	VPS4B	0.904942	0.545346
209	EIF1B	0.342641	0.545018
210	COQ6	0.300941	0.544556
211	GBF1	0.686954	0.541657
212	AP3D1	0.587976	0.538946
213	GSTZ1	0.950647	0.538736
214	DCK	0.169609	0.537991
215	NANS	0.540271	0.537778
216	STAT3	0.178277	0.536545
217	PMM2	0.076294	0.533729

218	FAM21A	0.02035	0.53272
219	AGPAT5	0.169228	0.532022
220	VAMP8	0.989945	0.531909
221	MSI2	0.741538	0.530232
222	PMVK	0.015374	0.528574
223	POLR3A	0.713437	0.528548
224	CTNND1	0.41401	0.527781
225	UROD	0.307069	0.527082
226	GALNS	0.699477	0.526042
227	PTK2	0.342047	0.523444
228	ROCK1	0.075674	0.523082
229	NAPRT	0.136506	0.52275
230	SMAP2	0.694681	0.522574
231	GLA	0.666253	0.522158
232	CARS	0.498014	0.521917
233	PDHB	0.576509	0.518002
234	GALNT2	0.301425	0.517985
235	PTPN1	0.485228	0.517425
236	RDX	0.074617	0.515981
237	LGMN	0.275564	0.51428
238	SNX5	0.010857	0.513622
239	TCERG1	0.371877	0.513559
240	CASP6	0.812432	0.511323
241	GNAI2	0.646242	0.511102
242	DNM1	0.612003	0.511091
243	USP7	0.870349	0.51109
244	ARHGEF7	0.627006	0.511075
245	RPIA	0.915377	0.50942
246	GSPT2	0.527311	0.508257
247	ADSL	0.477584	0.507975
248	ALG1	0.576454	0.505994
249	MTM1	0.758077	0.505978
250	SPTLC1	0.109956	0.505557
251	STUB1	0.54753	0.505124
252	PRDX6	0.208695	0.504973
253	EIF3G	0.251649	0.502817
254	APIP	0.122739	0.501523
255	RRM2B	0.21222	0.500053

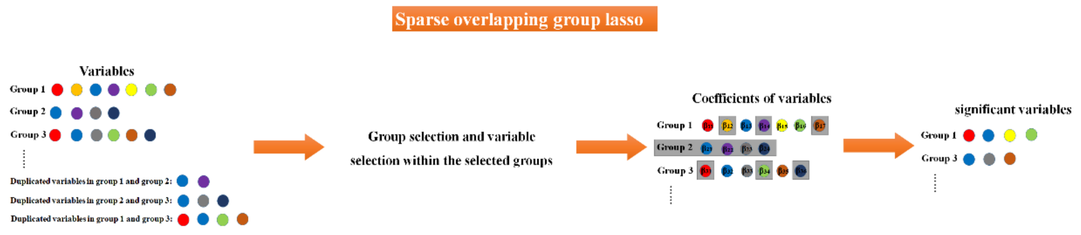

Supplementary Figure S1. Schematic diagram of sparse overlapping group lasso method. “Variables” includes a wide variety of data, such as genomics, proteomics, and metabolomics data. Shades of gray at the part of “Coefficients of variables” represented the coefficients of variables were estimated to zero. If all coefficients in a group were estimated to zero, the group had no effect on the outcomes and was eliminated.

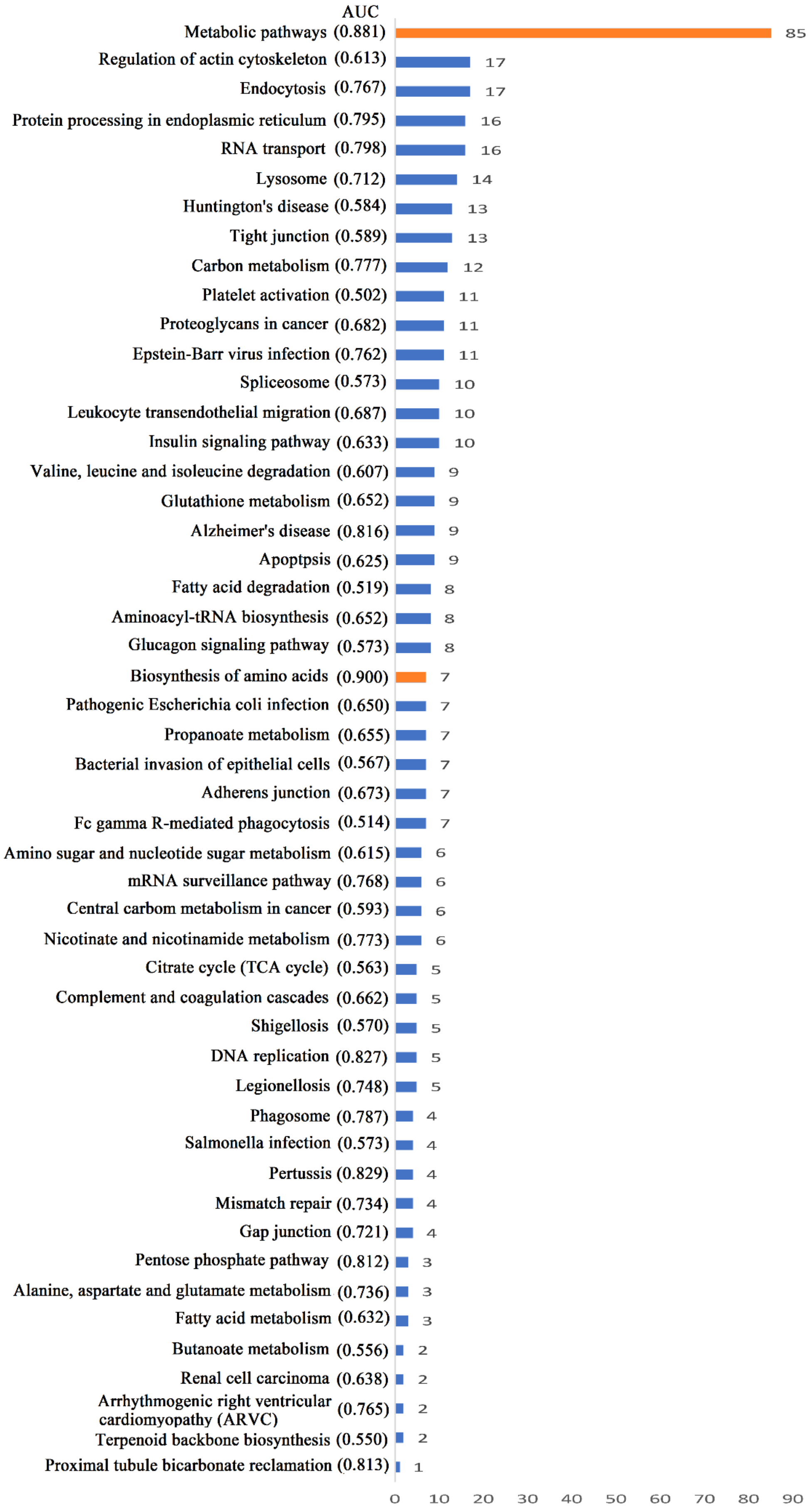

AUC
Metabolic pathways (0.881) 85
Regulation of actin cytoskeleton (0.613) 17
Endocytosis (0.767) 17
Protein processing in endoplasmic reticulum (0.795) 16
RNA transport (0.798) 16
Lysosome (0.712) 14
Huntington's disease (0.584) 13
Tight junction (0.589) 13
Carbon metabolism (0.777) 12
Platelet activation (0.502) 11
Proteoglycans in cancer (0.682) 11
Epstein-Barr virus infection (0.762) 11
Spliceosome (0.573) 10
Leukocyte transendothelial migration (0.687) 10
Insulin signaling pathway (0.633) 10
Valine, leucine and isoleucine degradation (0.607) 9
Glutathione metabolism (0.652) 9
Alzheimer's disease (0.816) 9
Apoptpsis (0.625) 9
Fatty acid degradation (0.519) 8
Aminoacyl-tRNA biosynthesis (0.652) 8
Glucagon signaling pathway (0.573) 8
Biosynthesis of amino acids (0.900) 7
Pathogenic Escherichia coli infection (0.650) 7
Propanoate metabolism (0.655) 7
Bacterial invasion of epithelial cells (0.567) 7
Adherens junction (0.673) 7
Fc gamma R-mediated phagocytosis (0.514) 7
Amino sugar and nucleotide sugar metabolism (0.615) 6
mRNA surveillance pathway (0.768) 6
Central carbom metabolism in cancer (0.593) 6
Nicotinate and nicotinamide metabolism (0.773) 6
Citrate cycle (TCA cycle) (0.563) 5
Complement and coagulation cascades (0.662) 5
Shigellosis (0.570) 5
DNA replication (0.827) 5
Legionellosis (0.748) 5
Phagosome (0.787) 4
Salmonella infection (0.573) 4
Pertussis (0.829) 4
Mismatch repair (0.734) 4
Gap junction (0.721) 4
Pentose phosphate pathway (0.812) 3
Alanine, aspartate and glutamate metabolism (0.736) 3
Fatty acid metabolism (0.632) 3
Butanoate metabolism (0.556) 2
Renal cell carcinoma (0.638) 2
Arrhythmogenic right ventricular cardiomyopathy (ARVC) (0.765) 2
Terpenoid backbone biosynthesis (0.550) 2
Proximal tubule bicarbonate reclamation (0.813) 1
0 10 20 30 40 50 60 70 80 90

Supplementary Figure S2. 50 pathways and 455 proteins that associated with OS of HGSC. The length of bar represented the number of selected proteins in each pathway. The orange bars were pathways had the predictive accuracy large than 0.85. Numerical value in bracket is the relative time-dependent AUC value for each pathway.

Data analysis scripts:

```
#import data
clinical_information<-read.csv("~/TCGA_protein/data_all/clinical information.csv",header=T)
TCGA_protein<-read.csv("~/TCGA_protein/data_all/TCGA_protein.csv",header=T)
proteinNames_left<-read.csv("~/TCGA_protein/data_all/protein name.csv")
proteinid_protein_map<-TCGA_protein[,1:2]
#data management
rownames(clinical_information)<-clinical_information[,1]
clinical_information<-clinical_information[,-1]
TCGA_protein_new<-TCGA_protein[TCGA_protein[,1] %in% proteinNames_left[,1],]
proteinid_protein_map<-TCGA_protein_new[,1:2]
TCGA_protein_new<-TCGA_protein_new[,-1]
TCGA_protein_new<-t(TCGA_protein_new)
colnames(TCGA_protein_new)<-TCGA_protein_new[1,]
TCGA_protein_new<-TCGA_protein_new[-1,]
rownames(TCGA_protein_new)<-substr(rownames(TCGA_protein_new),9,12)
rownames(clinical_information)<-substr(rownames(clinical_information),9,12)
#delete 5 samples in clinical information
clinical_information<-clinical_information[rownames(clinical_information) %in% rownames(TCGA_protein_new),]
data_analysis<-data.frame(clinical_information,TCGA_protein_new)
#enrichment analysis
protein_name<-colnames(data_analysis)[-c(1:15)]
library("org.Hs.eg.db")
library(clusterProfiler)
library(BiocGenerics)
entrezID <- mget(protein_name, org.Hs.egSYMBOL2EG, ifnotfound=NA)
entrezID <- as.character(entrezID)
protein_id_map<-cbind(protein_name,entrezID)
rownames(protein_id_map)<-protein_id_map[,2]
protein_id_map<-protein_id_map[,-2,drop=F]
proteinid_protein_id_map<-cbind(protein_id_map,as.character(proteinid_protein_map[,1]))
entrezID_delete<-entrezID[!entrezID=="NA"]
enrichKEGG<- enrichKEGG(gene = entrezID_delete,organism = "hsa",
pAdjustMethod="fdr",maxGSSize=20000,qvalueCutoff=0.05,pvalueCutoff = 0.05)
enrichKEGG_result1<-enrichKEGG@result
geneID<-enrichKEGG_result1[,8,drop=F]
colnames(TCGA_protein_new)<-entrezID
data_all_analysis<-matrix(NA,nrow(data_analysis),0)
index_all<-c()
pretein_name<-c()
index_description<-c()
protein_id<-c()
for (i in (1:nrow(geneID))){
```

```
  data_all_analysis<-cbind(data_all_analysis,TCGA_protein_new[,unlist(strsplit(geneID[i,],"/"))])
  pretein_name<-c(pretein_name,protein_id_map[unlist(strsplit(geneID[i,],"/")),1])
  index_all<-c(index_all,c(rep(i,length(unlist(strsplit(geneID[i,],"/"))))))
  index_description<-c(index_description,c(rep(enrichKEGG_result1[i,2],length(unlist(strsplit(geneID[i,],"/"))))))
  protein_id<-c(protein_id,proteinid_protein_map[unlist(strsplit(geneID[i,],"/")),1])
}
data_analysis$status[data_analysis$vital_status=="DECEASED"]<-1
data_analysis$status[data_analysis$vital_status=="LIVING"]<-0
data_all_analysis1<-matrix(as.numeric(data_all_analysis),nrow(data_all_analysis))
colnames(data_all_analysis1)<-colnames(data_all_analysis)
rownames(data_all_analysis1)<-rownames(data_all_analysis)
data_all_SGL=list(x=data_all_analysis1,time=as.matrix(round(as.numeric(data_analysis$daystodeath_or_LFU)/30,1)),status=as.factor(da
ta_analysis$status))
library(SGL)
set.seed(20161219)
TCGA_protein_all<-cvSGL(data_all_SGL,index_all,type="cox",verbose=T)
plot(TCGA_protein_JHU)
beta_fit<-TCGA_protein_all$fit
beta_all<-beta_fit$beta
logmin_best<-TCGA_protein_all$lldiff
a1<-min(logmin_best)
beta_all1<-cbind(index_description,colnames(data_all_analysis))
beta_all2<-cbind(beta_all1,beta_all)
beta_all3<-cbind(pretein_name,beta_all2)
beta_all3<-cbind(protein_id,beta_all3)
beta_all_select_all<-beta_all3[,c(1,2,3,4,24)]
beta_all_select<-beta_all_select_all[!beta_all_select_all[,5]==0,]
colnames(beta_all_select)<-c("protein_id","pretein_name","index_description","geneID","beta")
write.csv(beta_all_select,"~/TCGA_protein/data_all/selected pathway and protein.csv")
data_analysis$status[data_analysis$vital_status=="DECEASED"]<-1
data_analysis$status[data_analysis$vital_status=="LIVING"]<-0
data_analysis$time<-as.numeric(round(as.numeric(data_analysis$daystodeath_or_LFU)/30,1))
data_analysis_end<-data_analysis[,-c(1:15)]
###time-dependent AUC####
path_name_new<-unique(beta_all_select[,3])
path_name_new<-path_name_new[-31]
AUC_path_new<-c()
for (i in path_name_new){
  library(ROCt)
  temp<-as.matrix(TCGA_protein_new[,rownames(beta_all_select[beta_all_select[,3]==i,])])
  temp1<-matrix(as.numeric(temp),nrow(temp))
  colnames(temp1)<-colnames(temp)
  rownames(temp1)<-rownames(temp)
  time<-data_analysis$time
```

```
  status<-data_analysis$status
  PI<-as.matrix(temp1) %*% as.numeric(beta_all_select[beta_all_select[,3]== i,5])
  v.cut <- quantile(PI , probs=seq(0.1, 0.9, by=0.01))
  roc1 <- crude.ROCt(times=time,
                     failures=status, variable=PI,
                     pro.time=120, cut.off=v.cut, estimator="naive")
  cindex.orig<-roc1$auc
  AUC_path_new<-c(AUC_path_new,cindex.orig)
}
AUC_path_new_PI<-cbind(as.character(path_name_new),AUC_path_new)
write.csv(AUC_path_new_PI,"~/TCGA_protein/data_all/AUC_path_new_PI.csv")
#####Variable selection#####
variable_name_new<-unique(beta_all_select[,2])
all_select<-data_analysis[,colnames(data_analysis)%in%variable_name_new]
data_analysis$status[data_analysis$vital_status=="DECEASED"]<-1
data_analysis$status[data_analysis$vital_status=="LIVING"]<-0
data_analysis$time<-as.numeric(round(as.numeric(data_analysis$daystodeath_or_LFU)/30,1))
all_select_end<-data.frame(data_analysis$time,data_analysis$status,all_select)
colnames1<-c(colnames(all_select_end[,-1:-2]))
colnames2<-c("time","status")
colnames<-c(colnames2,colnames1)
colnames(all_select_end)<-colnames
write.csv(all_select_end,"~/TCGA_protein/data_all/all_select_end.csv")
all_select_end<-read.csv("~/TCGA_protein/data_all/all_select_end.csv",header=T,stringsAsFactors = FALSE)
all_select_end<-all_select_end[,-1]
library(survival)
coxwald_all<-numeric(ncol(all_select_end))
p_all<-numeric(ncol(all_select_end))
for (i in 3 : ncol(all_select_end)){
  coxwald_all[i]<-coxph(Surv(time,status)~ all_select_end[,i],data=all_select_end)$wald.test
  p_all[i]<-(1-pnorm(sqrt(coxwald_all[i])))*2
}
p_all<-data.frame(p_all)
#### Time-dependent AUC####
AUC_protein_all<-c()
cindex.orig<-c()
for (i in 3:ncol(all_select_end)){
  library(ROCt)
  v.cut <- quantile(all_select_end[,i] , probs=seq(0.1, 0.9, by=0.01))
  roc1 <- crude.ROCt(times=all_select_end$time,
                     failures=all_select_end$status, variable=all_select_end[,i],
                     pro.time=120, cut.off=v.cut, estimator="naive")
  cindex.orig<-roc1$auc
  #
```

```
    AUC_protein_all<-c(AUC_protein_all,cindex.orig)
}
names<-colnames(all_select_end)[-1:-2]
p_value<-p_all[-1:-2,]
all_select_result<-cbind(names,p_value,AUC_protein_all)
write.csv(all_select_result,"~/TCGA_protein/data_all/all_select_result.csv")
#####relationship #####
data_see_X<-cbind(all_select_end$EIF2B1,all_select_end$PRPS1L1,all_select_end$MAPK13,all_select_end$C3,
                  all_select_end$CFH,all_select_end$CFB,all_select_end$RHEB,all_select_end$FEN1,
                  all_select_end$OPLAH)
########## univariate time dependent AUC value>0.9 ###
data_all_Y<-all_select_end[,c(1,2)]
data_all_X<-cbind(all_select_end$EIF2B1,all_select_end$PRPS1L1,all_select_end$MAPK13)
data_all<-data.frame(data_all_Y,data_all_X)
library(survival)
fit<-coxph(Surv(time,status)~ ., data = data_all)
PI_all<-numeric(nrow(data_all))
for (i in 1:nrow(data_all)){
    PI_all[i]<--0.947*data_all$X1[i]-0.623*data_all$X2[i]-0.578*data_all$X3[i]
    }
PI_all
PI_all_cox<-data.frame(data_all_Y,PI_all)
fit_all<-coxph(Surv(time,status)~ ., data = PI_all_cox)###p=1.95e-05
####calculate AUC###
library(ROCt)
v.cut <- quantile(PI_all_cox$PI_all , probs=seq(0.1, 0.9, by=0.01))
roc_all <- crude.ROCt(times=PI_all_cox$time,
                      failures=PI_all_cox$status, variable=PI_all_cox$PI_all,
                      pro.time=120, cut.off=v.cut, estimator="naive")
cindex.orig_all<-roc_all$auc
plot(1-roc_all$table$sp, roc_all$table$se, ylim=c(0,1), xlim=c(0,1), ylab="sensitivity",
     xlab="1-specificity", type="l", lty=1, col=2, lwd=2)
abline(c(0,0), c(1,1), lty=2)
legend("bottomright", paste("OS, (AUC=", round(roc_all$auc, 3), ")", sep=""),
       lty=1, lwd=2, col=2)
######PFS #################################
data_analysis_recurrence<-data_analysis[data_analysis[,10]!="Not available",]
time_c<-c()
for ( i in 1:nrow(data_analysis_recurrence)){
    time_c[i]<-round(as.numeric(as.character(data_analysis_recurrence[i,10]))/30,1)
}
time_c
data_analysis_recurrence$status[data_analysis_recurrence$Prog_or_Recur=="Yes"]<-1
data_analysis_recurrence$status[data_analysis_recurrence$Prog_or_Recur=="No"]<-0
```

```
status_c=data_analysis_recurrence$status
data_all_PFS=data.frame(time_c,status_c,data_analysis_recurrence[,c(-1:-15)])
data_PFS_PRE<-data.frame(time_c,status_c,data_all_PFS$EIF2B1,data_all_PFS$PRPS1L1,data_all_PFS$MAPK13)
PSF_name<-c("time","status","EIF2B1","PRPS1L1","MAPK13")
colnames(data_PFS_PRE)<-PSF_name
data_PSF_Y<-data_PFS_PRE[,c(1,2)]
data_PFS_PRE$EIF2B1<-as.numeric(as.character(data_PFS_PRE$EIF2B1))
data_PFS_PRE$PRPS1L1<-as.numeric(as.character(data_PFS_PRE$PRPS1L1))
data_PFS_PRE$MAPK13<-as.numeric(as.character(data_PFS_PRE$MAPK13))
data_PSF_X<-cbind(data_PFS_PRE$EIF2B1,data_PFS_PRE$PRPS1L1,data_PFS_PRE$MAPK13)
data_PSF<-data.frame(data_PSF_Y,data_PSF_X)
library(survival)
fit<-coxph(Surv(time,status)~ ., data = data_PSF)
PI_PSF<-numeric(nrow(data_PSF))
for (i in 1:nrow(data_PSF)){
  PI_PSF[i]<--0.384*data_PSF$X1[i]-0.260*data_PSF$X2[i]-0.675*data_PSF$X3[i]
}
PI_PSF
PI_PSF_cox<-data.frame(data_PSF_Y,PI_PSF)
fit_PSF<-coxph(Surv(time,status)~ ., data = PI_PSF_cox)###P=0.00087
library(ROCt)
v.cut <- quantile(PI_PSF_cox$PI_PSF , probs=seq(0.1, 0.9, by=0.01))
roc_PSF <- crude.ROCt(times=PI_PSF_cox$time,
                      failures=PI_PSF_cox$status, variable=PI_PSF_cox$PI_PSF,
                      pro.time=120, cut.off=v.cut, estimator="naive")
cindex.orig_PSF<-roc_PSF$auc
plot(1-roc_PSF$table$sp, roc_PSF$table$se, ylim=c(0,1), xlim=c(0,1), ylab="sensitivity",
     xlab="1-specificity", type="l", lty=1, col=2, lwd=2)
abline(c(0,0), c(1,1), lty=2)
legend("bottomright", paste("PSF, (AUC=", round(roc_PSF$auc, 3), ")", sep=""),
       lty=1, lwd=2, col=2)
PI_all
PI_PSF
write.csv(PI_PSF_cox,"~/TCGA_protein/data_all/PI_PSF_cox.csv")
###multiple analysis OS###
data_multi1<-read.csv("~/TCGA_protein/data_all/ multiple analysis.csv",header=T)
library(survival)
fit_multi<-coxph(Surv(time,status)~ PI+ stage+platinum, data = data_multi1)
data_multi2<-read.csv("~/TCGA_protein/data_all/ multiple analysis.csv",header=T)
library(survival)
fit_multi<-coxph(Surv(time,status)~ PI_PSF+ stage+platinum, data = data_multi2)
##OS predictive accuracy####
data_multi_platinum<-data_multi1[,c(2,3,7,26)]
data_multi_platinum<-na.omit(data_multi_platinum)
```

```
library(ROCt)
v.cut <- quantile(data_multi_platinum$platinum , probs=seq(0.1, 0.9, by=0.01))
roc_PSF <- crude.ROCt(times=data_multi_platinum$time,
                        failures=data_multi_platinum$status, variable=data_multi_platinum$platinum,
                        pro.time=120, cut.off=v.cut, estimator="naive")
cindex.orig_PSF<-roc_PSF$auc
plot(roc_PSF$table$se, 1-roc_PSF$table$sp, ylim=c(0,1), xlim=c(0,1), ylab="sensitivity",
     xlab="1-specificity", type="l", lty=1, col=2, lwd=2)
abline(c(0,0), c(1,1), lty=2)
legend("bottomright", paste("pOS, (AUC=", round(1-roc_PSF$auc, 3), ")", sep=""),
       lty=1, lwd=2, col=2)
fit_multi<-coxph(Surv(time,status)~ PI+platinum, data = data_multi_platinum)

PI_P_OS<-numeric(nrow(data_multi_platinum))
for (i in 1:nrow(data_multi_platinum)){
  PI_P_OS[i]<-1.117*data_multi_platinum$PI[i]-1.705*data_multi_platinum$platinum[i]
}
PI_P_OS
PI_P_predic<-data.frame(data_multi_platinum[,1:2],PI_P_OS)
PI_P_predic
library(ROCt)
v.cut <- quantile(PI_P_predic$PI_P_OS , probs=seq(0.1, 0.9, by=0.01))
roc_PSF <- crude.ROCt(times=PI_P_predic$time,
                        failures=PI_P_predic$status, variable=PI_P_predic$PI_P_OS,
                        pro.time=120, cut.off=v.cut, estimator="naive")
cindex.orig_PSF<-roc_PSF$auc
plot(1-roc_PSF$table$sp, roc_PSF$table$se, ylim=c(0,1), xlim=c(0,1), ylab="sensitivity",
     xlab="1-specificity", type="l", lty=1, col=2, lwd=2)
abline(c(0,0), c(1,1), lty=2)
legend("bottomright", paste("p+pOS, (AUC=", round(roc_PSF$auc, 3), ")", sep=""),
       lty=1, lwd=2, col=2)
data_multi_platinum_pfs<-data_multi2[,c(2,3,4,20)]
data_multi_platinum_pfs<-na.omit(data_multi_platinum_pfs)
library(ROCt)
v.cut <- quantile(data_multi_platinum_pfs$platinum , probs=seq(0.1, 0.9, by=0.01))
roc_PSF <- crude.ROCt(times=data_multi_platinum_pfs$time,
                        failures=data_multi_platinum_pfs$status, variable=data_multi_platinum_pfs$platinum,
                        pro.time=120, cut.off=v.cut, estimator="naive")
cindex.orig_PSF<-roc_PSF$auc
plot(roc_PSF$table$se, 1-roc_PSF$table$sp, ylim=c(0,1), xlim=c(0,1), ylab="sensitivity",
     xlab="1-specificity", type="l", lty=1, col=2, lwd=2)
abline(c(0,0), c(1,1), lty=2)
legend("bottomright", paste("pPFS, (AUC=", round(1-roc_PSF$auc, 3), ")", sep=""),
       lty=1, lwd=2, col=2)
```

```
data_multi_platinum_pfs<-data_multi2[,c(2,3,4,17)]
data_multi_platinum_pfs<-na.omit(data_multi_platinum_pfs)
library(ROCt)
v.cut <- quantile(data_multi_platinum_pfs$stage , probs=seq(0.1, 0.9, by=0.01))
roc_PSF <- crude.ROCt(times=data_multi_platinum_pfs$time,
                      failures=data_multi_platinum_pfs$status, variable=data_multi_platinum_pfs$stage,
                      pro.time=120, cut.off=v.cut, estimator="naive")
cindex.orig_PSF<-roc_PSF$auc
plot(1-roc_PSF$table$sp,roc_PSF$table$se , ylim=c(0,1), xlim=c(0,1), ylab="sensitivity",
     xlab="1-specificity", type="l", lty=1, col=2, lwd=2)
abline(c(0,0), c(1,1), lty=2)
legend("bottomright", paste("sPFS, (AUC=", round(roc_PSF$auc, 3), ")", sep=""),
       lty=1, lwd=2, col=2)
data_multi_platinum_pfs<-data_multi2[,c(2,3,4,17,20)]
fit_multi<-coxph(Surv(time,status)~ PI_PSF+stage+platinum, data = data_multi_platinum_pfs)
PI_P_PFS<-numeric(nrow(data_multi_platinum_pfs))
for (i in 1:nrow(data_multi_platinum_pfs)){
  PI_P_PFS[i]<-0.8974*data_multi_platinum_pfs$PI_PSF[i]+0.4286*data_multi_platinum_pfs$stage[i]-
2.6748*data_multi_platinum_pfs$platinum[i]
}
PI_P_PFS
PI_P_predic_pfs<-data.frame(data_multi_platinum_pfs[,1:2],PI_P_PFS)
##PFS: PI+stage+platinum ####
PI_P_predic_pfs<-na.omit(PI_P_predic_pfs)
library(ROCt)
v.cut <- quantile(PI_P_predic_pfs$PI_P_PFS , probs=seq(0.1, 0.9, by=0.01))
roc_PSF <- crude.ROCt(times=PI_P_predic_pfs$time,
                      failures=PI_P_predic_pfs$status, variable=PI_P_predic_pfs$PI_P_PFS,
                      pro.time=120, cut.off=v.cut, estimator="naive")
cindex.orig_PSF<-roc_PSF$auc
plot(1-roc_PSF$table$sp, roc_PSF$table$se, ylim=c(0,1), xlim=c(0,1), ylab="sensitivity",
     xlab="1-specificity", type="l", lty=1, col=2, lwd=2)
abline(c(0,0), c(1,1), lty=2)
legend("bottomright", paste("p+p+sOS, (AUC=", round(roc_PSF$auc, 3), ")", sep=""),
       lty=1, lwd=2, col=2)
```
